# Control of Metabolic Homeostasis by Stress Signaling Is Mediated by the Lipocalin NLaz

**DOI:** 10.1371/journal.pgen.1000460

**Published:** 2009-04-24

**Authors:** Julie Hull-Thompson, Julien Muffat, Diego Sanchez, David W. Walker, Seymour Benzer, Maria D. Ganfornina, Heinrich Jasper

**Affiliations:** 1 Department of Biology, University of Rochester, Rochester, New York, United States of America; 2 Division of Biology, California Institute of Technology, Pasadena, California, United States of America; 3 Departamento de Bioquímica y Biología Molecular y Fisiología, Instituto de Biología y Genética Molecular, Universidad de Valladolid-CSIC, Valladolid, Spain; University of California San Francisco, United States of America

## Abstract

Metabolic homeostasis in metazoans is regulated by endocrine control of insulin/IGF signaling (IIS) activity. Stress and inflammatory signaling pathways—such as Jun-N-terminal Kinase (JNK) signaling—repress IIS, curtailing anabolic processes to promote stress tolerance and extend lifespan. While this interaction constitutes an adaptive response that allows managing energy resources under stress conditions, excessive JNK activity in adipose tissue of vertebrates has been found to cause insulin resistance, promoting type II diabetes. Thus, the interaction between JNK and IIS has to be tightly regulated to ensure proper metabolic adaptation to environmental challenges. Here, we identify a new regulatory mechanism by which JNK influences metabolism systemically. We show that JNK signaling is required for metabolic homeostasis in flies and that this function is mediated by the *Drosophila* Lipocalin family member Neural Lazarillo (NLaz), a homologue of vertebrate Apolipoprotein D (ApoD) and Retinol Binding Protein 4 (RBP4). Lipocalins are emerging as central regulators of peripheral insulin sensitivity and have been implicated in metabolic diseases. NLaz is transcriptionally regulated by JNK signaling and is required for JNK-mediated stress and starvation tolerance. Loss of NLaz function reduces stress resistance and lifespan, while its over-expression represses growth, promotes stress tolerance and extends lifespan—phenotypes that are consistent with reduced IIS activity. Accordingly, we find that NLaz represses IIS activity in larvae and adult flies. Our results show that JNK-NLaz signaling antagonizes IIS and is critical for metabolic adaptation of the organism to environmental challenges. The JNK pathway and Lipocalins are structurally and functionally conserved, suggesting that similar interactions represent an evolutionarily conserved system for the control of metabolic homeostasis.

## Author Summary

Metabolism of multicellular organisms has to adjust to environmental changes. Insulin signaling plays an important role in this regulation. Stress signals can repress Insulin signaling, curtailing growth to promote stress tolerance and extend lifespan. While this interaction allows managing energy resources under stress conditions, excessive JNK activity in adipose tissue of vertebrates has been found to promote type II diabetes. Thus, the interaction between stress and Insulin signaling has to be carefully regulated to ensure proper metabolic adaptation. Here, we identify a new regulatory mechanism by which stress signaling influences metabolism in fruitflies. We show that an evolutionarily conserved secreted protein, Neural Lazarillo (NLaz), is induced in response to stress signals, and that it is required for metabolic regulation. NLaz mutant animals are more sensitive to stress and show significant metabolic deficiencies. Similarly, increased expression of NLaz inhibits growth, but increases stress and starvation tolerance. We show that these functions are mediated by an interaction with the Insulin signaling pathway. Our results show that the regulation of NLaz by stress signals is critical for metabolic adaptation of the organism to environmental challenges. Both the Insulin and JNK signaling mechanisms analyzed here are evolutionarily conserved, suggesting that similar interactions control metabolic adaptation in vertebrates.

## Introduction

System-wide coordination of cellular energy consumption and storage is crucial to maintain metabolic homeostasis in multicellular organisms. It is becoming increasingly apparent that endocrine mechanisms that are required for this coordination impact the long-term health of adult animals and significantly influence lifespan and environmental stress tolerance [1]–[3]. Insulin/IGF signaling (IIS) is central to this regulation, as loss of Insulin signaling activity impairs metabolic homeostasis, but induces stress tolerance and increases lifespan in a variety of model organisms [1]–[3]. Interestingly, environmental stress and cellular damage can systemically repress IIS activity, suggesting the existence of adaptive response mechanisms by which metazoans manage energy resources in times of need [4]–[8]. The mechanism(s) and mediators of this endocrine regulatory system are only beginning to be understood [1]–[3],[9].

Studies in flies and worms have recently identified the stress-responsive Jun-N-terminal Kinase (JNK) signaling pathway as an important component of such an adaptive metabolic response to stress. JNK activation, which can be induced by a variety of environmental stressors, including oxidative stress, represses IIS activity, extending lifespan but limiting growth [4],[10]. Interestingly, similar effects of JNK signaling are also observed in mammals, in which it represses Insulin signal transduction by various mechanisms, including an inhibitory phosphorylation of Ser-307 of the insulin receptor substrate, as well as activation of the transcription factor FoxO [11],[12]. This inhibition contributes to Insulin resistance and the metabolic syndrome in obese mice [13], suggesting that chronic inflammatory processes (which result in activation of JNK signaling) are central to the etiology of metabolic diseases in obese individuals [9].

Endocrine interactions between Insulin-producing and various Insulin-responsive tissues are likely to coordinate the adaptive metabolic response described above [1]–[3]. JNK-mediated activation of Foxo in Insulin Producing Cells (IPCs) of flies, for example, represses the expression of insulin-like peptide 2 (dilp2), regulating growth and longevity [4]. At the same time, Foxo activation in the fatbody results in lifespan extension, presumably by an endocrine mechanism that feeds back to IPCs [14],[15].

Adipose tissue is increasingly being recognized as an important regulator of metabolic homeostasis. It secretes a variety of so-called adipokines, including the inflammatory cytokine TNF-alpha [9]. TNF-alpha activates JNK signaling, contributing to JNK-mediated insulin resistance in mouse models for obesity [9],[13]. JNK activation in adipose tissue further induces expression of IL-6, which specifically induces Insulin resistance in the liver [16]. While the chronic inhibition of insulin signaling by adipose-derived inflammatory cytokines thus has deleterious effects in obese individuals, it is likely that such endocrine interactions have evolved to govern metabolic homeostasis systemically in an adaptive manner [9]. Supporting this view, adipose tissue is an important regulator of lifespan in worms, flies, and mice, and it is emerging that systemic inhibition of Insulin signaling by adipose-derived factors is involved in this effect [1]–[3].

An endocrine role for adipose tissue in metabolic regulation has further been demonstrated in mice with adipose-specific deletion of the glucose transporter GLUT4, in which secretion of the Lipocalin family member RBP4 from fat cells induces insulin resistance throughout the organism [4],[17],[18]. Such an endocrine system is expected to be adaptive, since it preserves glucose for only the most essential functions during starvation or environmental stress. At the same time, mis-regulation of this system is likely to contribute to metabolic diseases like type II diabetes. Accordingly, increased serum levels of RBP4 are found in obese and diabetic individuals [19], and polymorphisms in the *rbp4* locus are associated with type II diabetes [20].

The Lipocalins are a large family of mostly secreted proteins that bind small hydrophobic ligands [21],[22]. Lipocalin family members are characterized by a low sequence similarity (reflecting diversification of biological functions), but a highly conserved tertiary protein structure and similar exon/intron structures of their genes [23],[24]. Recent studies implicate various Lipocalins in the regulation of systemic insulin action and of stress responses [18], [25]–[28]. Interestingly, the neuroprotective Lipocalin ApoD is strongly induced in aging mice, rhesus macaques and humans, suggesting evolutionarily conserved regulation of this gene [29], an it induces insulin resistance when overexpressed in the mouse brain [30].

The *Drosophila* genome contains three Lipocalin genes: *NLaz*, *GLaz*, and *karl*. Unlike the protein Lazarillo in more ancient insect lineages, which is GPI-anchored to the cell membrane of neurons [31], all *Drosophila* Lipocalins are secreted extracellular proteins, like ApoD and all other vertebrate Lipocalins. Recent studies have identified an important role for GLaz in stress resistance and lifespan control as well as in the regulation of lipid storage [32],[33]. While the function of NLaz remains unclear, *in situ* hybridization in *Drosophila* embryos shows that it is expressed in a subset of neuronal cells, and, interestingly, in the developing fat body [34], indicating a potential role in the systemic regulation of metabolism.

Here, we show that *NLaz* transcription is induced by oxidative stress and by JNK signaling in the fatbody, influencing metabolic homeostasis in the fly. Importantly, NLaz induces stress and starvation tolerance downstream of JNK signaling, and negatively regulates Insulin signaling, disrupting glucose homeostasis, repressing growth, and extending lifespan. Our results thus indicate that induction of NLaz mediates the antagonistic interaction between JNK and Insuling signaling in flies, acting as part of a stress response mechanism that adjusts metabolism and growth in response to environmental insults.

## Results

### JNK Signaling Is Required for Metabolic Homeostasis in Flies

**Figure 1:**
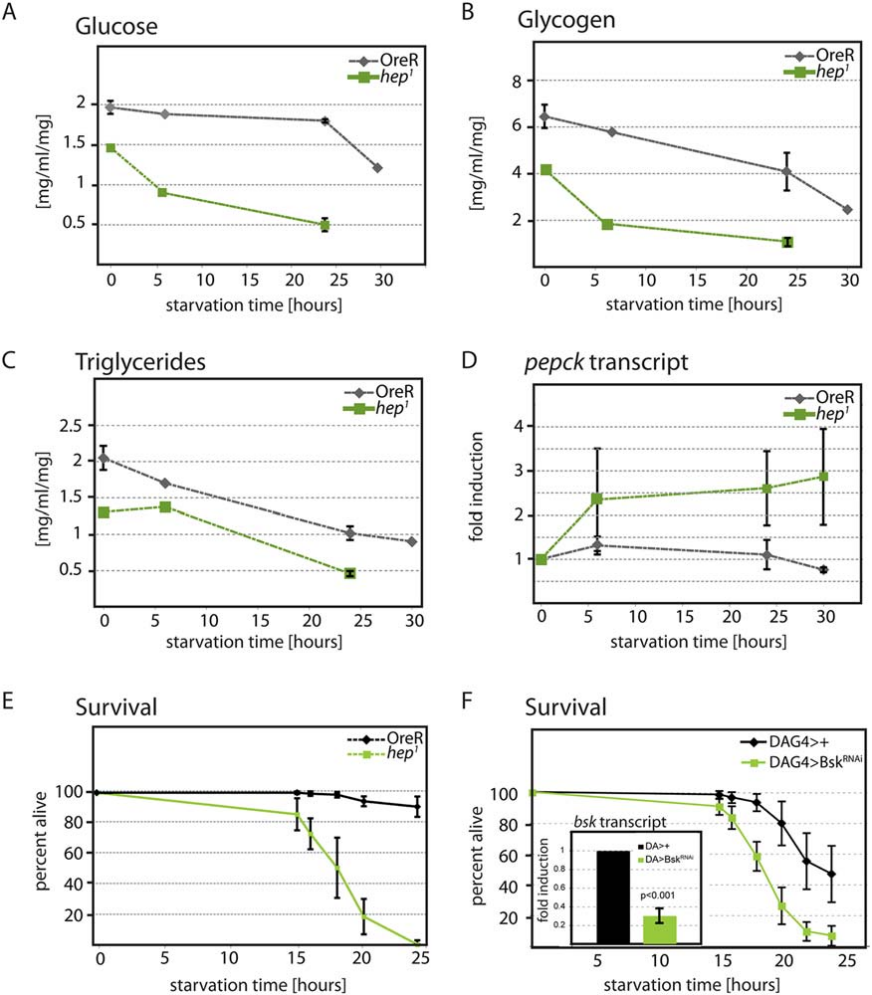
JNK is required for maintenance of metabolic homeostasis. (A–E) Comparison of hemizygous *hep^1^* males (*hep^1^/y*) to wild type OreR males. (A–C) Carbohydrate and lipid content in homogenates prepared from populations of 10 flies prior to and after 6, 24, and 30 hours of wet starvation. All measurements were normalized to the average weight of a single fly in its population. (A) Glucose. (B) Glycogen. (C) Triglycerides. (D) Real time PCR results from cDNA prepared from starved adult males collected 6, 24, and 30 hours after wet starvation. Levels of *PEPCK* are compared in starved conditions to fed controls (0 hours). All transcripts are normalized to *actin5C*. (E,F) Percent of male flies surviving after prolonged dry starvation. Population sizes were (E) OreR: n = 151; *hep^1^*: n = 103. Lifespan differences are statistically significant (p<0.001, log rank test). (F) DaG4/+: n = 179; BskRNAi/+;DaG4/+: n = 138. p<0.001, log rank test. (F, inset) Real time PCR on cDNA prepared from Bsk^RNAi^/+;DaG4/+ larvae. Levels of *Bsk* are compared to DAG4/+ controls. All transcripts are normalized to *rp49*.

Based on the ability of JNK signaling to antagonize IIS activity in flies and worms [4],[10], and on the starvation tolerance of flies with increased JNK signaling activity [35], we hypothesized that this pathway plays a role in regulating metabolic homeostasis under physiological conditions. To start characterizing such a role, we analyzed the maintenance of nutrient stores under starvation conditions in wild-type flies and in flies mutant for the JNK activating Kinase Hemipterous (JNKK/Hep). Interestingly, males hemizygous for the *hep* loss-of-function allele *hep^1^* exhibited significantly reduced energy stores (lipids and carbohydrates) in *ad libitum* conditions compared to wild-type control flies, suggesting impaired metabolic homeostasis in these animals (Figure 1A–C, Figure S1A). Accordingly, we found that in *hep^1^* mutants, nutrient stores were rapidly depleted upon starvation. Interestingly, *hep^1^* mutants also exhibited an accelerated and increased gluconeogenic response to starvation (Figure 1D), measured by *phosphoenolpyruvate carboxykinase* (PEPCK) expression [36], supporting the idea that JNK signaling mutants suffer a rapid decline in available free sugars upon starvation. Consistent with this view, *hep^1^* hemizygotes are significantly more sensitive to starvation than wild-type controls (Figures 1E and Figure S1B). Similarly, flies in which JNK signaling was repressed by ubiquitous over-expression of a dsRNA against the *Drosophila* JNK Basket (Bsk) were sensitive to starvation, confirming a loss of metabolic homeostasis in JNK loss-of-function conditions (Figure 1F and Figure S1C).

Interestingly, these findings recapitulate similar observations in IIS gain-of-function conditions, which lead to a decrease in steady-state metabolic stores and starvation sensitivity, as well as IIS loss-of-function conditions, which show the opposite results [37]–[41]. The effects of JNK signaling on metabolism thus support the perceived antagonism between JNK signaling and IIS in the regulation of energy homeostasis [4],[10]. Since we had previously found that JNK represses *dilp2* transcription in IPCs in response to oxidative stress ([4] and Karpac et al., *submitted*), we tested whether the starvation sensitivity in *hep^1^* mutants correlates with elevated *dilp2* transcription. Surprisingly, we found no difference in *dilp2* (nor *dilp3* or *dilp5*) transcript levels in *hep^1^* mutants compared to wild-type controls under *ad libitum* conditions, and no changes in *dilp2* transcription in response to starvation (Figure S1D, E, and data not shown; *dilp2* transcript levels are insensitive to nutritional conditions, see [42]). These results suggest that, while JNK regulates *dilp2* transcription to regulate systemic responses to stress, other targets of JNK might be mediating the control of metabolic homeostasis. Since mammalian JNK acts in adipose tissue to induce Insulin resistance, we focused on the fatbody as a potential site of action for JNK in flies.

### NLaz Transcription Is Induced in the Fatbody in Response to JNK Activation

**Figure 2:**
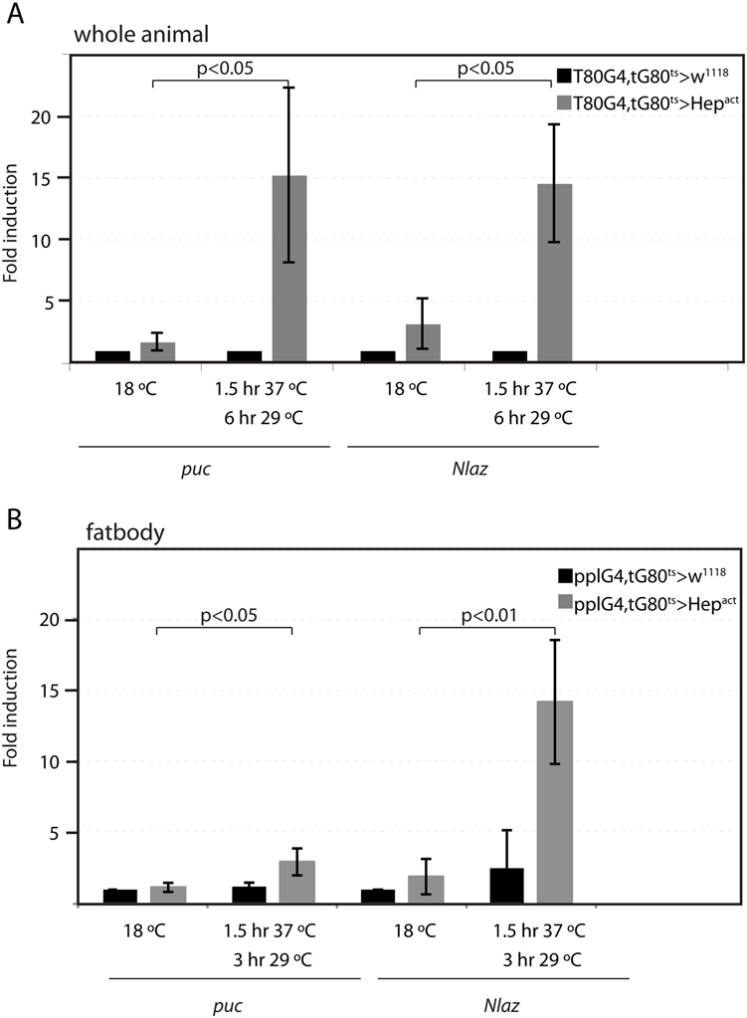
JNK regulates transcription of NLaz. (A, B) Real time PCR measuring transcript levels of *puc* and *NLaz* in whole larvae (A) and dissected fatbody (B). Larvae express Hep^act^ under the control of the ubiquitous T80-Gal4 driver (A) or the fatbody pplG4 driver (B) combined with tubGal80^ts^. Genotypes: (A) *w^1118^*; T80Gal4, tubGal80^ts^/UAS-Hep^act^; control genotype: *w^1118^*; T80Gal4, tubGal80^ts^/+; (B) *w^1118^*; pplGal4, tubGal80^ts^/UAS-Hep^act^; control genotype: *w^1118^*; pplGal4, tubGal80^ts^/+. Larvae were reared at 18°C until 96 hours after egg laying, heat shocked for 1.5 hours at 37°C and left at 29°C for 6 hours (A) or 3 hours (B) to activate the driver. Transcript levels are normalized to *rp49*. Averages and Standard Deviations of three independent experiments are shown. p values were calculated using Student's T test.

To identify potential mediators of JNK-induced metabolic changes, we tested the transcriptional response of a number of candidate genes to JNK activation. We focused on the putative adipokines and secreted regulators of Insulin signaling, dALS, IMP-L2, GLaz, Karl, and NLaz, since these molecules or their mammalian homologues have been implicated in systemically governing metabolic homeostasis. Activation of JNK was achieved by over-expression of a constitutively active Hep (Hep^act^) in larvae using the TARGET system, which allows heat-inducible expression of UAS-linked transgenes [43]. We expressed Hep^act^ ubiquitously (using the ubiquitous driver T80-Gal4), or specifically in the fatbody (using the fatbody driver ppl-Gal4, expressed both in larval and adult fatbody, [36] and Figure S8), for a short period of time, increasing the likelihood of observing direct transcriptional effects of increased JNK activity. Transcript levels of potential JNK target genes were then assessed by real-time RT-PCR (Figure 2 and Figure S2A).

Among the tested molecules, we found that transcription of the Lipocalin NLaz was potently induced in both whole larvae as well as specifically in the fatbody in response to JNK activation, within a timeframe that resembles the induction of *puc*, a *bona fide* JNK signaling target gene (Figure 2). Another Lipocalin, *Karl*, was also induced by JNK signaling, albeit to a lesser extent (Figure S2A) while GLaz was not induced (Figure S2A) suggesting differential regulation of *Drosophila* Lipocalins by JNK. Since JNK can activate the transcription factor Foxo, we also tested whether Foxo regulates NLaz transcription. We found that JNK still induces NLaz in a Foxo mutant background (Figure S2B) and that over-expression of constitutively active Foxo (FoxoTM) was not sufficient to induce NLaz (Figure S2C). NLaz thus appears to be a Foxo-independent downstream effector of JNK signaling.

### NLaz Acts Downstream of JNK to Maintain Metabolic Homeostasis

**Figure 3:**
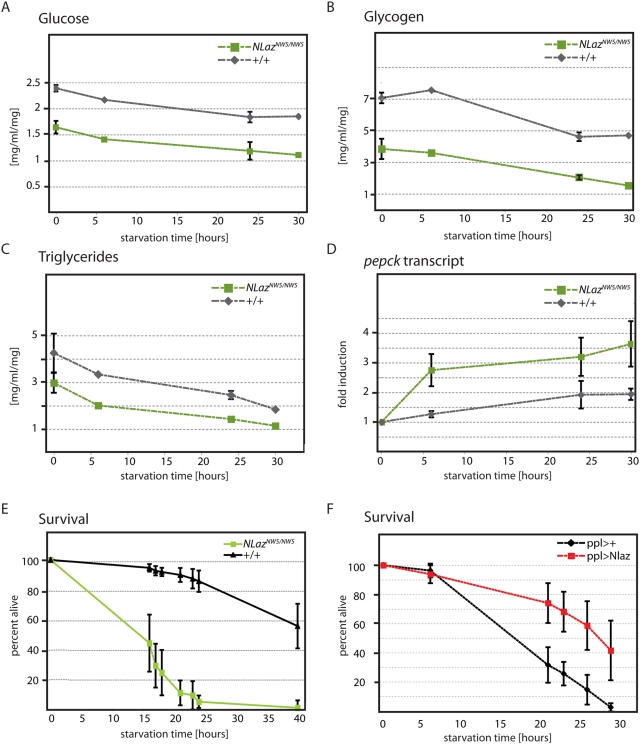
NLaz is required for maintenance of metabolic homeostasis. (A–E) Comparison of homozygous NLaz loss of function mutants (NLaz*^NW5/NW5^*) to wild type isogenic controls (*NLaz^C-NW-14/C-NW-14^*). (A–C) Carbohydrate and lipid content in homogenates prepared from populations of 10 male flies prior to and after 6, 24, and 30 hours of wet starvation. All measurements were normalized to the average weight of a single fly in its population. (A) Glucose (B) Glycogen (C) Triglycerides. (D) Real time RT-PCR results from cDNA prepared from starved adult males collected 6, 24, and 30 hours after wet starvation. Levels of *PEPCK* are compared in starved conditions to fed controls (0 hours). All transcripts are normalized to *actin5C*. (E,F) Percent of male flies surviving after being exposed to prolonged dry starvation. Genotypes: (E) *NLaz^CNW14/CNW14^*: n = 227; *NLaz^NW5/NW5^*: n = 254. p<0.001, log rank test. (F) *pplG4/+*;*+/+*: n = 102; *pplG4/+*;UASNLaz/+: n = 115. p<0.001, log rank test. To equalize culture conditions and genetic backgrounds, sibling F1 progeny derived from out-crossed w^1118^; pplGal4/pplGal4 with out-crossed w^1118^; UAS-NLaz/+ are compared.

These results, and the known effects of the NLaz homologues ApoD and Rbp4 in mice [18],[30], suggest that NLaz or Karl might act downstream of JNK signaling to regulate metabolic homeostasis. To test this hypothesis, we measured carbohydrate and lipid levels in homozygous NLaz mutant flies (using the knock-out allele *NLaz^NW5^*, derived from the *NLaz^SceI^* allele [44]) and isogenic controls. Similar to *hep^1^* mutants, *NLaz* mutants exhibited reduced stores and rapid starvation-induced decline of glucose, trehalose, glycogen, and triglyceride levels (Figure 3A–C and Figure S2D). Likewise, NLaz mutants showed an accelerated gluconeogenic response (induction of PEPCK, [36]) and were sensitive to starvation (Figure 3D, E).

Since NLaz is induced in response to starvation (Figure S2E), and is expressed in the fatbody (Figure 2B and [34]), we tested whether NLaz or Karl over-expression in the fatbody would be sufficient to protect the organism from starvation sensitivity. Indeed, we found that expression of NLaz using ppl-Gal4 promotes starvation tolerance (Figure 3F and Figure S2E, F). In males, overexpression of NLaz results in increased glycogen stores while lipid levels decrease slightly and glucose levels remain normal (Figure S9), suggesting a shift in energy storage from lipids to glycogen. In females, on the other hand, Glycogen, Glucose and Lipids are increased when NLaz is overexpressed, demonstrating increased energy stores, accompanied by hyperglycemia (Figure S9). Expression of Karl, on the other hand, did not protect against starvation (Figure S3A), suggesting that *Drosophila* Lipocalins, similar to vertebrate Lipocalins, are functionally specialized [21],[22],[30].

**Figure 4:**
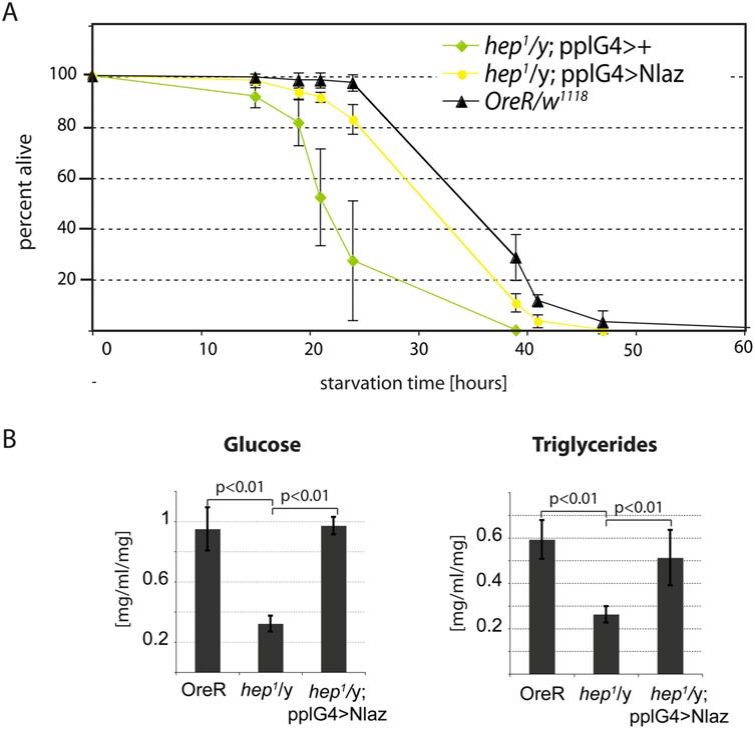
Nlaz acts downstream of JNK to maintain metabolic homeostasis. (A) Percent of male flies surviving after being exposed to prolonged dry starvation. Populations are F1 progeny of crosses between hep^1^/FM6; pplG4/CyO females and OreR males (hep^1^/y; pplG4/+; n = 65) or w^1118^; UAS-NLaz/TM3 males (hep^1^/y; pplG4/UASNlaz; n = 70). Wild-type controls are F1 progeny of OreR females crossed to w^1118^ males (OreR/w^1118^; n = 181). p<0.001 (log rank test) for difference between hep1/y; ppl/+ and either of the other two populations. p = 0.629 (log rank test) for hep1/y; pplG4/UASNLaz compared to OreR/w^1118^. (B) Glucose and lipid content in homogenates prepared from populations of 10 male flies after 24 hours of wet starvation.

These results suggested that JNK-mediated induction of NLaz in the fatbody regulates metabolic homeostasis. Supporting this view, we found that fatbody expression of NLaz was sufficient to restore starvation resistance and Glucose and Triglyceride levels in *hep^1^* mutants (Figure 4). Fatbody-derived NLaz is likely to be secreted (see [34], and Figure S7), serving as systemic regulator of metabolic homeostasis. Supporting such a systemic role, we found that expression of NLaz in other tissues, such as pericardial cells and hemocytes of flies (using the dorothy-Gal4 driver, dot-Gal4 [45]), also protects against starvation (Figure S3B). A localized supply of NLaz is thus sufficient to exert its systemic protective function.

### NLaz Promotes Defense Against Oxidative Stress, but not Against Infection

**Figure 5:**
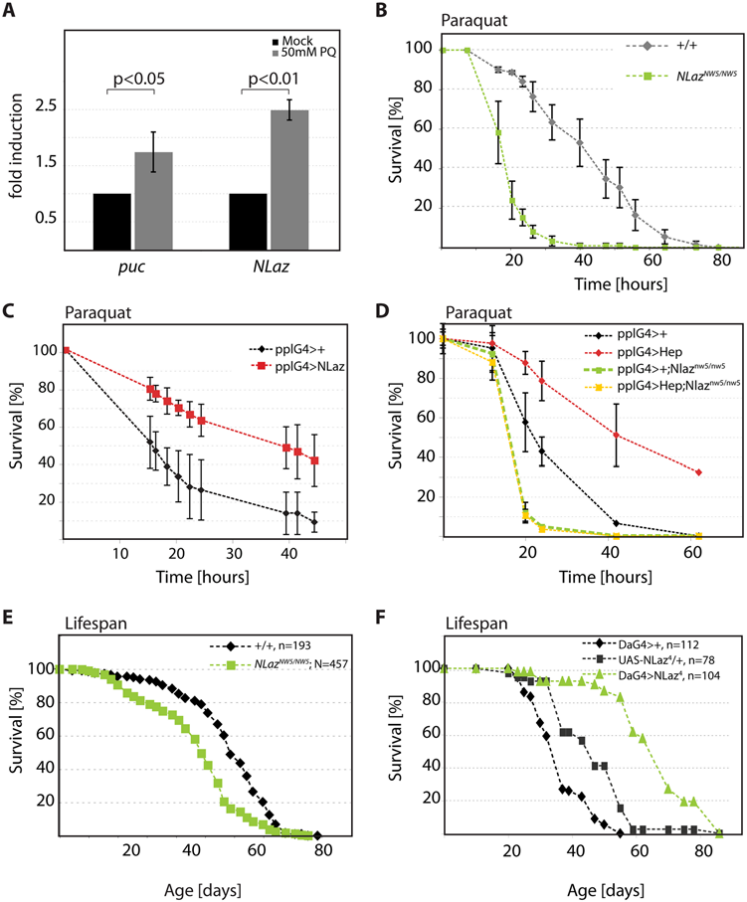
NLaz acts downstream of JNK to promote oxidative stress tolerance. (A) NLaz is induced in flies exposed to oxidative stress. Levels of *NLaz* are compared using cDNA prepared from wild type OreR flies fed 50 mM paraquat in 5% sucrose to those fed 5% sucrose alone for 24 hours. Levels of *puc* are shown for comparison. All transcript levels are normalized to *actin5C*. p values are calculated using Student's T test. (B) Oxidative stress sensitivity in NLaz mutants. Survival after exposure to paraquat. NLaz^CNW14/CNW14^ N = 70, NLaz^NW5/NW5^ N = 117. Log rank test, *p*<0.001. (C) Over-expressing UAS-NLaz using the ppl-Gal4 driver promotes tolerance to paraquat. Comparison of siblings of the following genotypes: *pplG4/+*, n = 103; pplG4/+;UASNLaz/+, n = 134. p<0.001(log rank test). (D) Stress sensitivity of NLaz mutants cannot be improved by JNK activation. Survival after exposure to paraquat. Populations of the following genotypes and numbers of individuals were used: *pplG4/+* n = 61; *pplG4/UASHep n = 97*; *pplG4,NW5/NW5 n = 135*; *pplG4,NW5/UASHep,NW5 n = 59*. Log rank test for pplG4/+ vs. pplG4/UASHep: p<0.001. (E and F) NLaz genedose influences lifespan. (E) Male longevity at 25°C. Groups of 20 flies per vial. NLaz^CNW14/CNW14^ N = 193, NLaz^NW5/NW5^ N = 457. Log-rank test, *p*<0.001. (F) Overexpression of NLaz increases normal survival of males at 25°C. Overexpressing *UAS-NLaz^4^* using *DaG4* as a ubiquitous driver increases mean and maximum life spans in normal conditions. DaG4/+, n = 112; UAS-NLaz^4^/+, n = 78; DaG4/UAS-NLaz^4^, n = 104; Log-rank test: p<0.001 (comparing UAS-NLaz and DaG4/UAS-NLaz).

JNK signaling promotes oxidative stress resistance in flies and worms. Similarly, reduced IIS activity also leads to stress tolerance, and the known crosstalk between these two pathways indicates that metabolic and stress responses are tightly linked, allowing organisms to balance protective and growth responses in accordance with available resources [1]–[3],[46]. Based on these studies and on our findings described above, we reasoned that NLaz-mediated metabolic changes can influence stress tolerance of flies. To test this hypothesis, we first assessed whether NLaz transcription would be induced in response to oxidative stress, and found that NLaz expression is indeed elevated in flies exposed to the reactive-oxygen inducing compound Paraquat (resembling the induction of *puc*; Figure 5A). We further tested stress sensitivity of NLaz mutants, and found that these flies exhibit increased sensitivity to Paraquat compared to wild-type control flies (Figure 5B). Over-expression of NLaz both ubiquitously and in the fatbody, on the other hand, confers resistance to Paraquat as well as hyperoxic conditions, supporting a protective role for NLaz in the fly (Figure 5C and Figure S4).

To test whether this function of NLaz was specific for oxidative stress, we assessed the sensitivity of NLaz over-expressing flies to infection with *Enterococcus faecalis*. *E. faecalis* infection is lethal to flies, but increased JNK as well as reduced IIS activity result in increased survival after infection [47]. Interestingly, NLaz over-expression in hemocytes (which mediate immune responses in flies; [48],[49]) was not sufficient to promote defense against infection with *E.faecalis* (Figure S4C), suggesting that NLaz acts specifically to protect against oxidative stress. Karl expression in hemocytes, on the other hand, is both sufficient and required for defense against *E. faecalis* infection (Figure S4D). These results, in addition to the differential effect of these two Lipocalins on starvation tolerance suggest that NLaz and Karl control distinct and specific physiological responses downstream of JNK signaling.

### JNK Activity in the Fatbody Confers NLaz-Dependent Oxidative Stress Tolerance

Stress protection by JNK signaling has been observed in *puc^E69^* mutants, which exhibit elevated JNK signaling throughout the organism [35], but also in flies over-expressing JNKK/Hep in neuronal tissue exclusively. This suggests that secreted molecules promote stress tolerance downstream of JNK signaling [4],[10]. One endocrine mechanism by which JNK activity promotes stress tolerance is downregulation of *dilp2* expression in IPCs ([4] and Hull-Thompson et al., in preparation). Interestingly, however, increased oxidative stress tolerance can also be observed when JNKK/Hep is specifically over-expressed in the fatbody (Figure 5D). To test whether NLaz induction might mediate the endocrine effects of fatbody-specific JNK activation, we assessed stress tolerance of NLaz mutant flies in which JNKK/Hep was over-expressed in the fatbody. Remarkably, we found that lack of NLaz completely abolished the ability of JNKK/Hep expression to promote oxidative stress tolerance (Figure 5D). These results indicate that Nlaz induction is an integral component of the JNK-mediated adaptation to environmental stress in *Drosophila*.

### NLaz Promotes Longevity

Our results thus support a model in which Nlaz is required downstream of JNK signaling to promote metabolic homeostasis and tolerance to certain forms of stress. Since elevated JNK signaling is associated with increased longevity in the fly, we tested the effect of NLaz on lifespan. Consistent with our other findings, flies lacking NLaz function are short-lived relative to isogenic controls (Figure 5E), while over-expression of NLaz with a ubiquitous driver increases lifespan (Figure 5F and Figure S6). These results further support the view that the effects of NLaz on metabolic homeostasis and stress tolerance are an important adaptive mechanism to preserve energy resources and optimize the fitness of the organism.

### NLaz Represses Growth and Affects Hemolymph Glucose Levels by Antagonizing IIS

Interestingly, the phenotypes we observed in flies over-expressing NLaz (starvation tolerance, higher nutrient stores, oxidative stress resistance and extended lifespan) are also phenotypes associated with reduced IIS activity in the fly [39]–[41], suggesting that the effects of NLaz might be mediated by inhibition of IIS.

**Figure 6:**
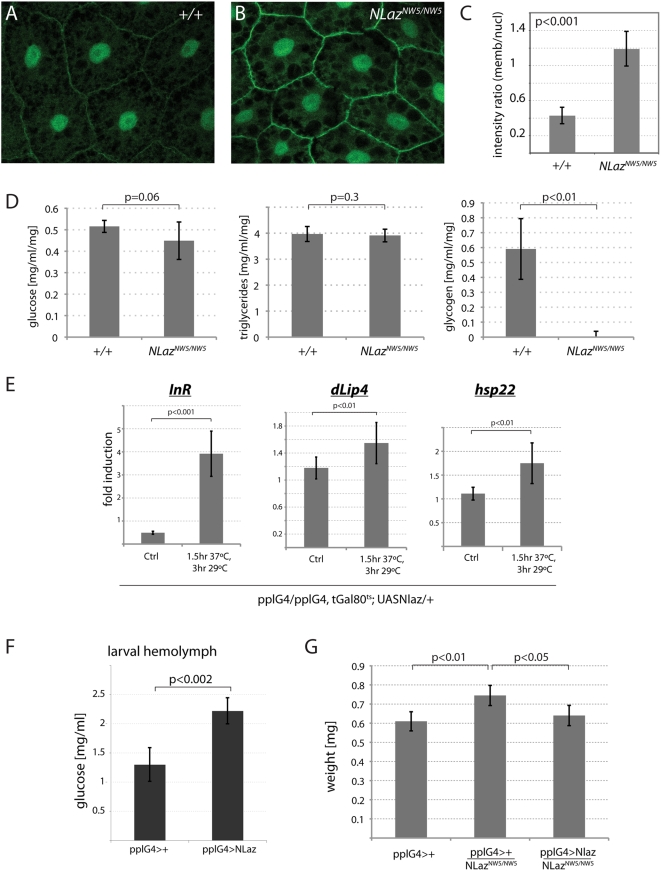
NLaz antagonizes IIS in larvae. (A–C) tGPH fluorescence [50] in larval fatbodies. (A) *NLaz^CNW14/CNW14^*. (B) *NLaz^NW5/NW5^*. Membrane localization of tGPH is increased in NLaz mutants compared to isogenic wild-type controls, indicating elevated PI3K activity. (C) Ratios of average membrane vs. nuclear fluorescence as determined using NIH ImageJ on images of fatbody cells of independent individuals. n = 6 for *NLaz^CNW14/CNW14^*, n = 10 for *NLaz^NW5/NW5^*. P value from Student's T test. (D) NLaz mutant third-instar larvae (homozygotes for *NLaz^NW5/NW5^*) exhibit strongly decreased Glycogen stores compared to isogenic wild-type controls (*NLaz^CNW14/CNW14^*). Glucose and Triglyceride levels do not differ in wild-type or NLaz mutant larvae. (E) Established Foxo target genes are induced in response to NLaz overexpression in the larval fatbody. Real-time PCR was performed to quantify dInR, dLip4, and hsp22 transcript levels in extracts of whole third-instar larvae. Transcript levels were normalized to *actin5C* and ratios of transcript levels in NLaz expressing larvae (pplG4/pplG4,tubGal80ts; UASNLaz/+) and in wildtype controls (pplG4/pplG4, tubGal80ts; +/+) are shown for control conditions (18°C) and after heat-shock. p-values from Student's Ttest. (F) Over-expression of NLaz in the fatbody results in elevated hemolymph glucose levels. Experiments were performed in larvae of the following genotypes: *pplG4/+*;*+/+*; *pplG4/+*; *UASNlaz/+*. (G) Comparison of adult sibling males of the following genotypes, reared at 29°C: pplG4; +/+. *pplG4,NLazNW5/NW5;+/+*; *pplG4,NLazNW5/NW5;UASNlaz/+*. Overall weight is increased in NLaz mutants. Size is decreased again in animals over-expressing NLaz in the fatbody. Fresh weight of males of the indicated genotypes.

To test this notion, we asked whether NLaz would affect other IIS-regulated processes in the fly. Indeed, we found elevated membrane localization of the reporter for PI3K activity, GFP-PH, in fatbody cells of NLaz mutant larvae, suggesting that Insulin signaling is increased in these cells in the absence of NLaz (Figure 6A–C; PI3K activity is indicative of Insulin signaling activity in flies and GFP-PH is widely used as a reliable reporter for this activity [50]). These larvae exhibited similar, albeit less general metabolic defects as adult NLaz mutants, with unaffected Glucose and Triglyceride levels, but complete loss of Glycogen stores (Figure 6D).

To further test whether NLaz influences IIS activity, we measured the expression of selected Foxo target genes in response to inducible NLaz over-expression in larval fatbodies. For this analysis, we selected genes that are established Foxo target genes in Drosophila, namely *thor*, *InR*, *dnaPolj*, *dLip4*, *hsp22*, *and l(2)efl* [4], [51]–[53]. A subset of these genes, *InR*, *dLip4* and *hsp22* showed significant changes in expression after induction of NLaz in the fatbody, indicating reduced IIS activity in these larvae (Figure 6E).

Further supporting a role for NLaz in repressing Insulin signaling, increased expression of NLaz in the fatbody also resulted in elevated hemolymph glucose levels in third-instar larvae (Figure 6F), a phenotype that is associated with reduced IIS activity and that is reminiscent of defects in Glucose homeostasis observed in Insulin resistant vertebrates [41]. NLaz gene dose and expression in the fatbody further moderately affected overall size of the animal, resulting in larger animals when NLaz is mutated, and rescue of this overgrowth when NLaz is overexpressed in the fatbody of NLaz mutant flies (Figure 6G).

**Figure 7:**
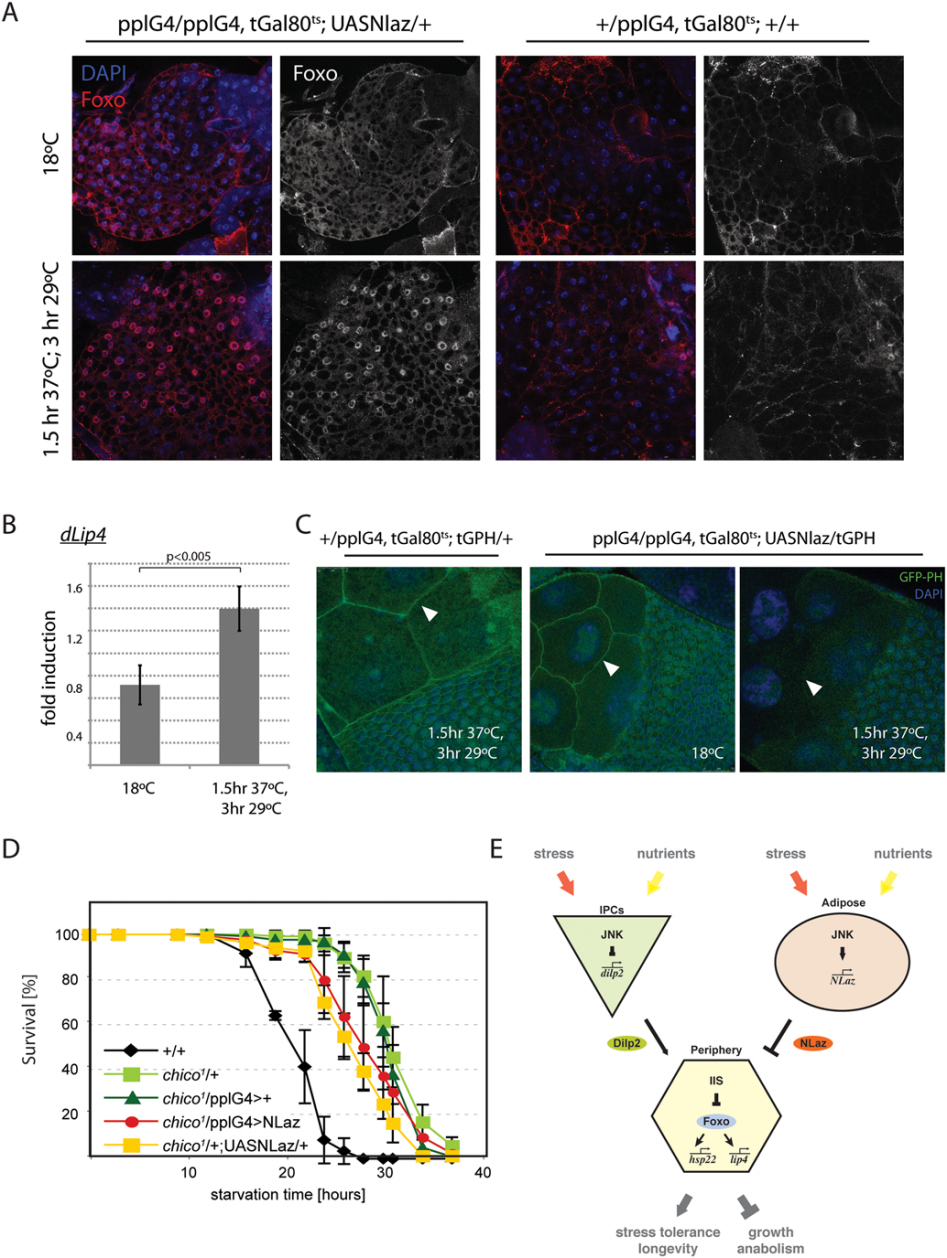
NLaz antagonizes IIS in adults. (A) Foxo translocates to the nucleus in adult fatbody tissue in response to NLaz induction. Confocal images of adult female fatbodies immustained for Foxo (red). DNA is visualized using DAPI (blue in the overlay). The monochrome panels show only Foxo signal. Adult flies (females and males, females are shown here) of the indicated genotypes were reared at 18°C and heat-shocked as indicated. (B) The Foxo target gene *dLio4* is induced in response to NLaz induction. Real-time PCR was performed to quantify dLip4 transcript levels in extracts of whole adult male NLaz-expressing flies and in wildtype controls (Genotypes as in A). Transcript levels were normalized to *actin5C* and ratio of transcript levels in NLaz expressing vs wild-type flies is shown for control conditions (18°C) and after heat-shock. P-value from Student's Ttest. (C) Fatbody-specific expression of NLaz induces changes in tGPH levels in nurse cells. Egg chambers of flies of the indicated genotypes are shown, tGPH fluorescence was determined by confocal microscopy. GFP-PH levels at the Nurse Cell boundaries are reduced in NLaz expressing flies after heatshock (arrowheads). (D) NLaz cannot promote further starvation tolerance of *chico1* mutant flies. Percent survival of males of the following genotypes in response to prolonged dry starvation: *+/+*, n = 112; *chico/+*, n = 124, *chico/pplG4*, n = 122; *chico/pplG4;UASNLaz/+*, n = 124; *chico/+;UASNLaz/+*, n = 78. Log rank: p<0.001 for all populations compared to +/+. (E) Proposed role of NLaz in promoting stress tolerance and metabolic homeostasis in the fly. JNK-mediated induction of NLaz in the fatbody is required for regulation of metabolic homeostasis and stress tolerance. Accordingly, NLaz over-expression promotes stress tolerance and extends lifespan. As suggested by our data and reported for related vertebrate Lipocalins, NLaz might interfere with Insulin signaling activity, thus coordinating metabolic changes throughout the organism. At the same time, JNK represses transcription of *dilp2* in IPCs. Together, these two mechanisms antagonize IIS in the periphery, thereby promoting stress tolerance systemically.

Together, these results suggest that fatbody-specific NLaz expression negatively regulates IIS activity in larvae. To test whether this effect of NLaz might contribute to the stress resistance and long lifespan observed in the adult, we assessed whether NLaz expression would also repress IIS activity in the adult fly. Indeed, induction of NLaz expression resulted in translocation of Foxo into the nucleus of fat body cells, suggesting decreased IIS activity (Figure 7A). This was accompanied by the induction of the Foxo target gene *Lip4*, but not other Foxo target genes, suggesting a context-dependent specific regulation of Foxo target genes in this background (Figure 7B).

To assess whether fatbody-specific expression of NLaz would affect IIS activity in other tissues, we observed GFP-PH fluorescence in the adult ovary, using GFP-PH localization in nurse cells as indicators for peripheral IIS activity. Indeed, NLaz expression resulted in significantly decreased GFP-PH signal at the cell membrane of nurse cells, further demonstrating negative regulation of IIS activity by secreted NLaz from the fatbody (Figure 6C).

The notion that NLaz acts upstream of IIS in metabolic regulation was also supported by the finding that over-expression of NLaz could not further promote starvation resistance of heterozygotes for the *chico* loss-of-function allele *chico^1^* (Figure 7D). Chico is the *Drosophila* homologue of Insulin receptor substrates, and *chico^1^* heterozygotes exhibit decreased IIS activity, and are stress and starvation tolerant [38],[39]. Expression analysis further indicates that *NLaz* acts downstream of insulin-like peptides, but upstream of *chico*, as *NLaz* expression is unaffected in *chico^1^* heterozygous animals (Figure S5B), and transcript levels of the three major insulin-like peptides, *dilp2*, *dilp3*, and *dilp5*, were unaffected by NLaz loss-or gain-of-function conditions, or by over-expression of Hep in the fatbody (Figure S5C, D). The repression of IIS by NLaz is thus not mediated by regulation of *dilp* transcription, but by downstream events that promote insulin resistance.

Taken together, these results support the notion that NLaz represses Insulin signaling systemically both in larvae and in adult flies.

## Discussion

Our findings support a role for JNK – mediated NLaz induction in the fatbody as a central part of an adaptive endocrine system that coordinates metabolism in response to environmental stress by regulating insulin sensitivity of peripheral tissues (Figure 7E). Recent studies have highlighted the role of adipose-derived endocrine factors in such adaptive responses [3],[9]. For example, reducing IIS activity or over-expressing Foxo specifically in adipose tissue leads to lifespan extension and stress tolerance in flies, mice and worms, presumably mediated by systemic repression of IIS [14],[15],[54],[55]. Furthermore, amino acid deprivation of *Drosophila* fat body cells leads to marked decreases in PI3K activity in wing imaginal discs and in the epidermis [56]. In vertebrates, on the other hand, excessive JNK activation in adipose tissue induces insulin resistance in the periphery, promoting Type II diabetes [16],[46],[57]. Our results implicate NLaz as a mediator of such systemic repression of IIS activity by adipose tissue.

JNK-mediated repression of IIS in flies is thus not only mediated by its function in IPCs, where it represses *dilp2* transcription (Wang et al., 2005; Hull-Thompson et al., in preparation), but also by adipose-specific induction of NLaz, which then inhibits IIS activity in insulin target tissues. This dual antagonism of IIS by JNK is intriguing, as it indicates that adaptive regulation of metabolism requires coordinated control of both insulin-like peptide production and peripheral insulin sensitivity (Figure 7E). How the relative contribution of these effects regulates the organism's metabolic homeostasis, stress resistance and lifespan, is an interesting question that will require further investigation.

Vertebrate Lipocalins have also been implicated in the modulation of insulin action, and recent studies suggest a protective role of these molecules under diverse stress conditions [18], [26]–[28],[30]. This function of Lipocalins thus emerges as an evolutionarily conserved adaptive mechanism, and our work integrates this mechanism into the known antagonism between JNK and IIS. Based on the evolutionary conservation of this antagonism it is tempting to speculate that vertebrate Lipocalins also act as effectors of JNK in the regulation of systemic insulin sensitivity, with important implications for potential therapeutic targeting of these molecules.

While generally promoting metabolic homeostasis and stress tolerance, functional specialization of different Lipocalin family members is expected due to their high sequence divergence. Accordingly, our data show that the Lipocalins present in *Drosophila* differ in regulation and function. While NLaz and GLaz both regulate stress sensitivity, only NLaz was found to be regulated by JNK signaling. Regulation of Karl, on the other hand, does not influence starvation tolerance (as NLaz does), but promotes resistance against infection by *E. faecalis*. Further investigation of this diversification of Lipocalin function promises to provide important insight into the systemic regulation of adaptation to diverse environmental challenges. Of particular interest will be to assess the role of Karl as a potential regulator of IIS during infection. Infection with Mycobacterium Marinum can result in significant repression of IIS activity, leading to phenotypes similar to wasting disease [58]. It is intriguing to speculate that excessive JNK-induced Karl expression may cause this pathology.

In humans, dysregulation of Lipocalins has been correlated with obesity, insulin resistance, and type II diabetes [18],[25],[59]. The cause for this mis-regulation of Lipocalin expression remains unclear, however. Our results implicate JNK signaling, which is activated chronically in obese conditions, as a possible cause. The finding that mammalian lipocalin-2, which impairs insulin action, is induced by the JNK activator TNFalpha, is especially intriguing [26]. Additional studies in vertebrates, as well as in the *Drosophila* model, will provide further insight into the physiological role of Lipocalins, their regulation by stress signaling, as well as their interaction with Insulin signaling. As Lipocalins are secreted molecules that bind hydrophobic ligands, it is further crucial to identify their physiological ligands in an effort to understand the mechanism(s) by which IIS activity is antagonized by Lipocalins. Such insight promises to provide a deeper understanding of the coordination of metabolic adaptation in metazoans as well as of the etiology of diabetes and other metabolic diseases.

## Materials and Methods

### Fly Lines and Handling

Fly lines were obtained as follows: OreR and Da-Gal4 from Bloomington stock center; hep1/FM6, gift from S. Noselli; ppl-Gal4, [36], gift from Michael Pankratz; dot-Gal4, gift from W.X. Li; UAS-Hep, gift from M. Mlodzik; UAS-bsk^RNAi^, from Vienna *Drosophila* RNAi Center (Transformant ID 34138). All fly lines used in this paper were tested for Wolbachia infection and found to be negative for Wolbachia.

Generation of the NLaz deletion strain (NLaz^SceI^) is described in [44]. Isogenic knock-out and control lines were generated by outcrossing NLaz^SceI^ knock-out flies into a *w^1118^*-CS_10_ wild type strain. Sister lines containing either the wild type allele of NLaz (line NLaz^CNW14^) or the mutant allele (line NLaz^NW5^) were selected by PCR and subsequent SceI restriction digest. The line *w^1118^*-CS_10_ is a 10 generations outcross of *w^1118^* into the CS background.

P-element mediated transformation of *w^1118^* mutant flies was used to generate pUASt-NLaz. The full-length NLaz cDNA was amplified using PCR (as annotated in flybase), and it was inserted into pUASt via ligation into EcoRI and XbaI sites. Three different independent insertion lines of pUASt-NLaz were used in our experiments, producing identical results. Flies were fed a cornmeal and molasses based diet, and were reared at 25°C. For each experiment, care was taken to ensure flies developed at an equal larval density.

### Starvation Tolerance and Nutrient Measurement

Starvation experiments were performed by placing flies in empty vials or vials with water-soaked filters, as indicated in figure legends. For glucose, trehalose, glycogen, and triglyceride measurements, cohorts of 10 male flies were weighed prior to homogenization in 100 μL homogenization buffer (0.01 M KH_2_PO_4_, 1 mM EDTA, pH 7.4). Homogenates were spun for 2 min. at 3,000 rpm, and the supernatant was collected. 10 μL of homogenate was used in each of the following assays: ***Glucose***: Homogenate was pipetted into 500 μL glucose reagent (Glucose (HK) Assay Kit, Sigma). Samples were incubated at room temperature for 15 min. and absorbance was measured at 340 nm versus deionized water. ***Trehalose***: 0.5 μL Trehalase from porcine kidney (Sigma) was added to homogenate. After 1 hour incubation at 37°C, glucose was measured as above, and the concentration of glucose prior to trehalose digestion was subtracted. ***Glycogen***: 10 μL of starch assay reagent (Starch assay kit, Sigma) was mixed with homogenate. Samples were shaken at 60°C for 15 minutes. 10 μL of sample was used in glucose assay already described. Absorbance of glucose prior to digestion with starch assay reagent was subtracted from final absorbance. ***Triglycerides***: Homogenate was added to 500 μL of activated triglyceride reagent (Liquicolor Triglycerides, Stanbio) and reaction was incubated at room temperature for 10 minutes. The absorbance was measured at 500 nm relative to activated triglyceride reagent. All metabolic measurements were normalized to fly weight.

### Paraquat and Hyperoxia Treatments

Flies were starved for 3–6 hours prior to re-feeding with 25 mM or 50 mM paraquat (Methyl Viologen, Sigma) in 5% sucrose. Paraquat solution was administered on soaked filters. Flies were kept in the dark once re-fed.

For hyperoxia, 20–30 2-day old adult males, were maintained in vials containing standard food within a Plexiglas enclosure of 28×28×24 inches at room temperature (22–24°C). Oxygen (100%) was passed through the box at a constant rate of 300 ml/min. Survival was assayed daily.

### Lifespan Analysis

Flies were collected within 24 hr of eclosion and were separated by sex at 2–3 days of age in groups of 20. They were raised at 25°C under a 12 hr∶12 hr light cycle and transferred to fresh food vials every 2–3 days.

To ensure identical genetic backgrounds, *Da-Gal4* was out-crossed 10 times to *w^1118^*. *Similarly*, UAS-*NLaz^4^* and UAS-*NLaz^8^* were generated and maintained in the *w^1118^* background, minimizing effects of genetic background variations and hybrid vigor in the progeny of the crosses studied. Remaining effects of genetic background and hybrid vigor can be assessed in lifespan differences between the progenies of the two control crosses (UAS-NLaz×w^1118^ and Da-GAL4×w^1118^). Any GAL4-dependent modification of lifespan observed in the test cross (Da-GAL4×UAS-NLaz), is thus due to the over-expression of the transgene.

### Real Time PCR

RNA was prepared from whole flies or larvae using Trizol reagent (Invitrogen) according to package instructions. Subsequently, Superscript reverse transcriptase (Invitrogen) and oligodT were used to generate cDNA. cDNA, diluted 1∶100, served as template for real time PCR using SYBR green based detection on a BioRad MyIQ thermal cycler. All reactions were performed in triplicate, and melting curves were examined to ensure single products. Quantification was performed using the “delta-delta Ct” method to normalize to actin5C or rp49 transcript levels and to control genotypes. Average Ct values of technical replicates were used for normalization, and all data presented here are averages and standard-deviations from at least three independent experiments. Primer pairs utilized were as follows:

*rp49*: 5′-CGGCACTCGCACATCATT, 5′-AGCTGTCGCACAAATGGC;
*actin5C*: 5′-CTCGCCACTTGCGTTTACAGT, 5′-TCCATATCGTCCCAGTTGGTC;
*puc*: 5′-CGAGGATGGGTTTGATTACGA, 5′-TCAGTCCCTCGTCAAATTGCT;
*NLaz*: 5′-GCCAGAAGTAGAACGGATACCA, 5′-ACTGGTGCAGCTGTAGACGAC;
*pepck*: 5′-CCAGGACAATTGCGGTCTGT, 5′-CTGCAGCATCCATGTCGCT;
*dLip4*: 5′-TGGATAGCTCAGCCACTT, 5′-GCGGGTATATCATGCTTTCC;
*hsp22*: 5′-CCAATCATATTCACCTGCCG, 5′-CATTCCAGGAGCCTTTGTAG;

### Quantification of tGPH Membrane Intensity

Nuclear fluorescence was used to normalize membrane fluorescence. Fat bodies of 5 individual wandering third instar larvae were imaged using confocal microscopy and average fluorescence of membrane and nuclei was measured using the histogram function of NIH ImageJ. Intensity ratios were calculated for n = 6–10 individual cells from different fatbodies, and overall averages and standard deviations were calculated.

### Weight and Wing Measurements

Sibling flies were used for comparisons. Flies were weighed on a Mettler Toledo Ultrafine balance as cohorts of 10 flies in pre-weighed eppendorf tubes. Wings were photographed, and size was determined by quantifying the number of pixels within each wing with adobe Photoshop.

### Transfection of S2 Cells and Detection of Secreted Proteins

S2 cells were maintained as adherent cultures at room temperature in Schneider's medium supplemented with 10% FBS. Cells were transfected with the pAHW plasmid (Murphy Laboratory), into which the Lipocalin cDNA had been subcloned using gateway recombination, fusing the protein with a C-terminal 3xHA tag. Regulation of trafficking of the fusion protein by the N-terminal signal sequence was thus maintained. Transfection was performed with the Fugene HD (Roche) reagent in a 9∶2 ratio per the manufacturer's instructions.

S2 cells were transfected with this construct and allowed to express the protein for 48 hours. The transfection medium was removed, and new medium was conditioned for 6 hours. Cells were separated from the conditioned medium by centrifugation (10′, 5000 rpm), and lysed in standard lysis buffer with protease inhibitors. An aliquot of the cell lysate and one from the conditioned medium were assayed for protein concentration (BCA assay, Pierce), and each adjusted to 1 ug/uL. 100 uL of these protein samples were mixed with SDS sample buffer, and denatured by heating to 95°C for 5 minutes. 10 ug of total protein from each sample were run on a denaturing SDS-page gel (Nupage, Invitrogen), and transferred to PVDF membrane following standard protocols. The membrane was then probed for HA reactivity using an HRP-conjugated anti-HA antibody (Roche), and detected using ECL West Dura substrate (Pierce).

## Supporting Information

Figure S1 (A) Trehalose content in homogenates prepared from populations of 10 flies prior to and after 6, 24, and 30 hours of wet starvation. (A) *hep1* and *+/+*. All measurements were normalized to the average weight of a single fly in its population. (B) Starvation sensitivity of hep^1^ mutant males in various genetic backgrounds. Males are progeny of crosses of hep^1^/FM6 to OreR (yellow n = 45), hep^1^/FM6 to w^1118^ (green n = 48) and hep^1^/FM6; pplG4/CyO to OreR (pink n = 40). Wild-type controls are progeny of OreR crossed to w^1118^. (C) Flies carrying the UAS-bsk^RNAi^ transgene in a wild-type background are not starvation sensitive. Sibling populations of progeny from crosses between w^1118^; UAS-bsk^RNAi^/+ to w^1118^ are shown (w^1118^; +/+: n = 67, w^1118^; UAS-bsk^RNAi^/+: n = 74). (D) *dilp2*, *dilp3 and dilp5* transcript levels in *hep^1^* mutants relative to *+/+* controls determined by real time RT-PCR. All transcript levels were normalized to *actin5C*. No significant differences are observed in transcript levels of ILPs between *hep^1^* and wild-type in *ad libitum* conditions. (E) No differences are observed in *dilp2* transcript levels in starved flies.
(0.7 MB TIF)

Figure S2 (A) Real time RT-PCR to measure changes in transcription of selected potential secreted metabolic regulators in response to JNK activation. The heat shock-inducible TARGET system was utilized. Average fold induction (between Hep^act^ expressing and wild-type controls) of non-heat shocked (reared at 18 degrees) and heat shocked samples are shown. Actin5C expression was used for normalization. (B) Transcriptional response of NLaz to JNK activation is not dependent on Foxo. Real time RT-PCR to measure changes in transcription of NLaz in larvae in which Hep^act^ is over-expressed using the TARGET system. Reducing the Foxo genedose (*dfoxo^25^* is a loss-of-function allele of *dfoxo*) does not affect the level of NLaz induction. (C) Over-expression of constitutively active Foxo (FoxoTM) is not sufficient to induce NLaz transcription. (D) Trehalose content of *NLaz^NW5/NW5^* compared to isogenic wild-type controls. (E) Flies carrying the UAS-NLaz transgene in a wild-type background are not starvation sensitive. Sibling populations of progeny from crosses between w^1118^;; UAS-NLaz/+ to w^1118^ are shown (w^1118^;; UAS-NLaz/+; n = 76, w^1118^;; +/+; n = 72). (F) Flies carrying the ppl-Gal4 transgene in a wild-type background are not starvation sensitive. Sibling populations of progeny from crosses between w1118; pplG4/+ to w1118; pplG4/+ are shown (w^1118^; pplG4/+; n = 66, w^1118^; pplG4/pplG4; n = 36; w^1118^; +/+; n = 36). (G) NLaz is induced in response to starvation in wild-types flies. NLaz transcript levels in adults of two wild-type strains (w1118 and CantonS) were measured by qRT-PCR. Prolonged (20 hr) starvation results in moderate increase of NLaz transcript.
(0.5 MB TIF)

Figure S3 (A, B) Percent survival in response to dry starvation. Genotypes: (A) pplG4/+, n = 181, pplG4/+;UASKarl/+, n = 181. (B) *hep1/y*, n = 134, +/+, n = 130, hep1/y;DorothyG4/+;UASNLaz/+, n = 46.
(0.4 MB TIF)

Figure S4 (A) Over-expression of NLaz enhances resistance to hyperoxia. Overexpressing *UAS-NLaz^4^ and UAS-NLaz^8^*, using *DaG4* as a ubiquitous driver protects from 100% oxygen-induced mortality. DaG4/+, n = 157; UAS-NLaz^4^/+, n = 106; DaG4/UAS-NLaz^4^, n = 84; UAS-NLaz^8^/+, n = 104; DaG4/UAS-NLaz^8^, n = 107. Log rank test for UAS-NLaz4: p<0.001. Log rank test for UAS-NLaz^8^: p<0.001. (B) Overexpressing *UAS-NLaz* using *DaG4* protects from paraquat-induced mortality. UAS-NLaz^4^ and UAS-NLaz^8^ are independent insertion lines of the same construct. DaG4/+, n = 118; UAS-NLaz^4^/+, n = 102; DaG4/UAS-NLaz^4^, n = 132; UAS-NLaz^8^/+, n = 94; DaG4/UAS-NLaz^8^, n = 116. Log rank test comparing UAS-NLaz4 and DaG4/UAS-NLaz^4^: p<0.001. Log rank test for UAS-NLaz^8^: p<0.001. (C, D) Percent survival in response to infection with *E. faecalis*. Genotypes: (C). *DorothyG4/+* mock, n = 21, *DorothyG4/+*, n = 111; *DorothyG4/+;UASNLaz/+*, n = 108. (D) *DorothyG4/+*, mock, n = 31; *DorothyG4/+*, n = 84; *DorothyG4/+*; *UASKarl/+*, n = 60, *DorothyG4/+;pWizKarl/+*, n = 56. Only initial mortality was recorded here, as flies that escape initial mortality live for at least another 2–3 weeks.
(0.6 MB TIF)

Figure S5 (A) Real time RT-PCR demonstrates that *NLaz* transcript levels are unchanged in *chico1* heterozygous mutants. (B) Real time RT-PCR measuring levels of *dilp2*, *dilp3*, and *dilp5* in cDNA prepared from dissected larval brains. Larval genotypes were as follows: *+/+*; *NLaz^NW5/NW5^*; *pplG4/+*; *pplG4/+*;*UASNLaz/+*; *pplG4/UASHep*. Transcript levels were normalized to *Actin5C*. (C) Real time RT-PCR measuring levels of *dilp2*, *dilp3*, and *dilp5* in adult heads from flies of the following genotypes: *+/+*, *NLaz^NW5/NW5^ pplG4/+*, *pplG4/+*;*UASNLaz/+*, *pplG4/UASHep*. All transcript levels are normalized to *Actin5C*.
(0.6 MB TIF)

Figure S6 NLaz over-expression from an alternative transgenic line promotes longevity. Overexpressing *UAS-NLaz^8^*, using *DaG4* as a ubiquitous driver, increases mean and maximum lifespans in normal conditions.UAS-NLaz^8^/+, n = 108; DaG4/UAS-NLaz^8^, n = 92. Log-rank test: p<0.001.
(0.2 MB TIF)

Figure S7 NLaz is secreted. HA-tagged NLaz (lanes 1 and 2) can be detected in the medium of S2 cells after 6 hrs of conditioning. Cell pellet (P) and supernatant (S) are shown. Related lipocalins are also secreted: human ApoD (lanes 3 and 4) and *Drosophila* GLaz (lanes 5 and 6).
(0.3 MB TIF)

Figure S8 pplG4 is active in the adult fatbody. GFP fluorescence can be observed throughout the body of male and female adult flies when pplG4 is used to drive UAS-nlsGFP expression. This signal is derived from the head and abdominal fatbodies. Dissected abdominal fatbody is shown in the third panel. Ppl does not drive expression in ovaries (compare fluorescence in fatbody attached to cuticle and ovaries).
(6.0 MB TIF)

Figure S9 Overexpression of NLaz affects metabolites in males and females. Glucose, Glycogen and Lipid levels in adult flies over-expressing NLaz under the control of pplGal4. Flies were reared at 25°C on normal food and metabolites were measured at 5 days of age.
(0.4 MB TIF)
