# Supplementary figures and images for: Control of Metabolic Homeostasis by Stress Signaling Is Mediated by the Lipocalin NLaz
Source: PLoS Genet. 2009 Apr 24;5(4):e1000460. doi: 10.1371/journal.pgen.1000460 (PMC2667264; doi:10.1371/journal.pgen.1000460)

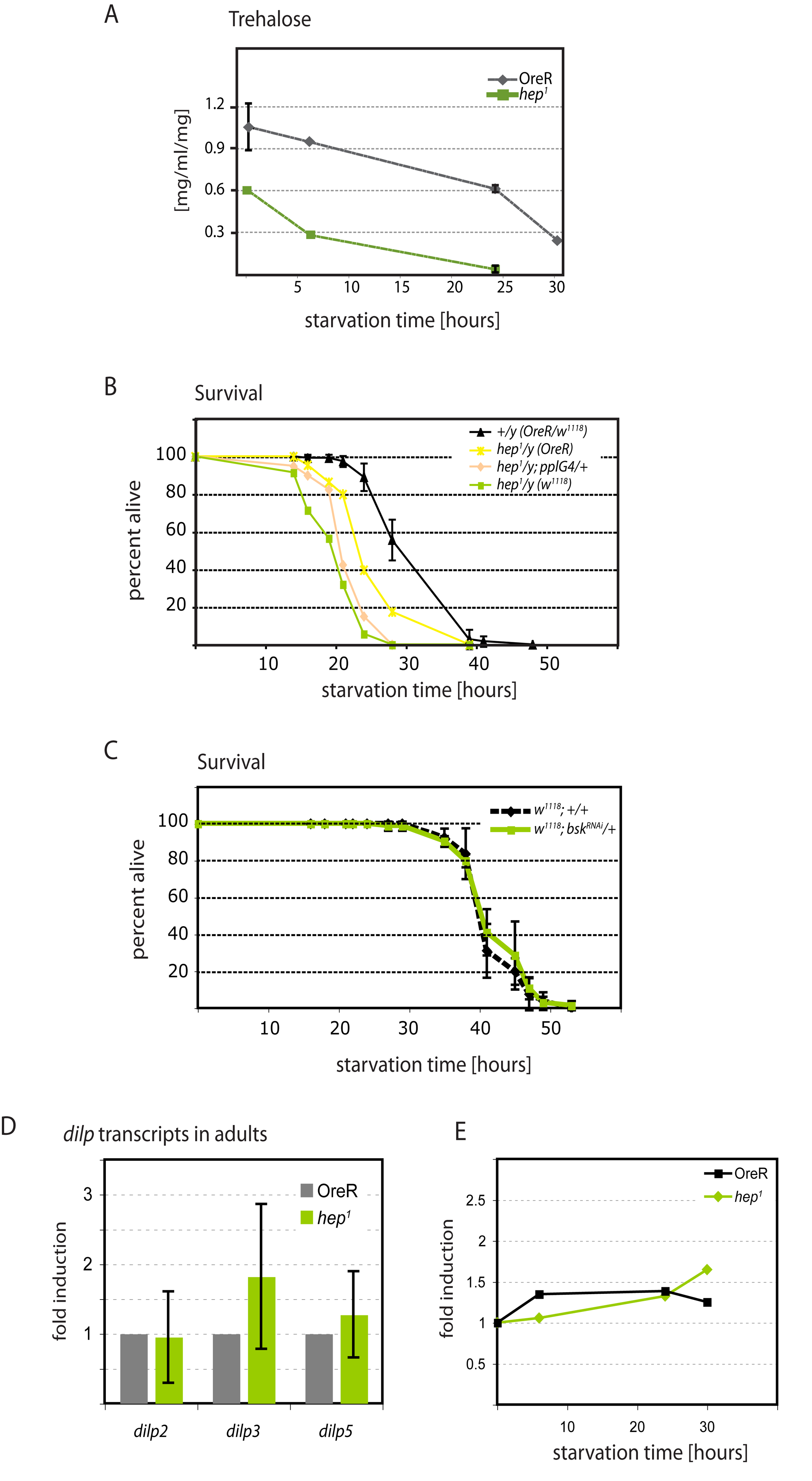

Supplement: Figure S1 — (A) Trehalose content in homogenates prepared from populations of 10 flies prior to and after 6, 24, and 30 hours of wet starvation. (A) hep1 and +/+. All measurements were normalized to the average weight of a single fly in its population. (B) Starvation sensitivity of hep1 mutant males in various genetic backgrounds. Males are progeny of crosses of hep1/FM6 to OreR (yellow n = 45), hep1/FM6 to w1118 (green n = 48) and hep1/FM6; pplG4/CyO to OreR (pink n = 40). Wild-type controls are progeny of OreR crossed to w1118. (C) Flies carrying the UAS-bskRNAi transgene in a wild-type background are not starvation sensitive. Sibling populations of progeny from crosses between w1118; UAS-bskRNAi/+ to w1118 are shown (w1118; +/+: n = 67, w1118; UAS-bskRNAi/+: n = 74). (D) dilp2, dilp3 and dilp5 transcript levels in hep1 mutants relative to +/+ controls determined by real time RT-PCR. All transcript levels were normalized to actin5C. No significant differences are observed in transcript levels of ILPs between hep1 and wild-type in ad libitum conditions. (E) No differences are observed in dilp2 transcript levels in starved flies. (0.7 MB TIF) [file pgen.1000460.s001.tif]

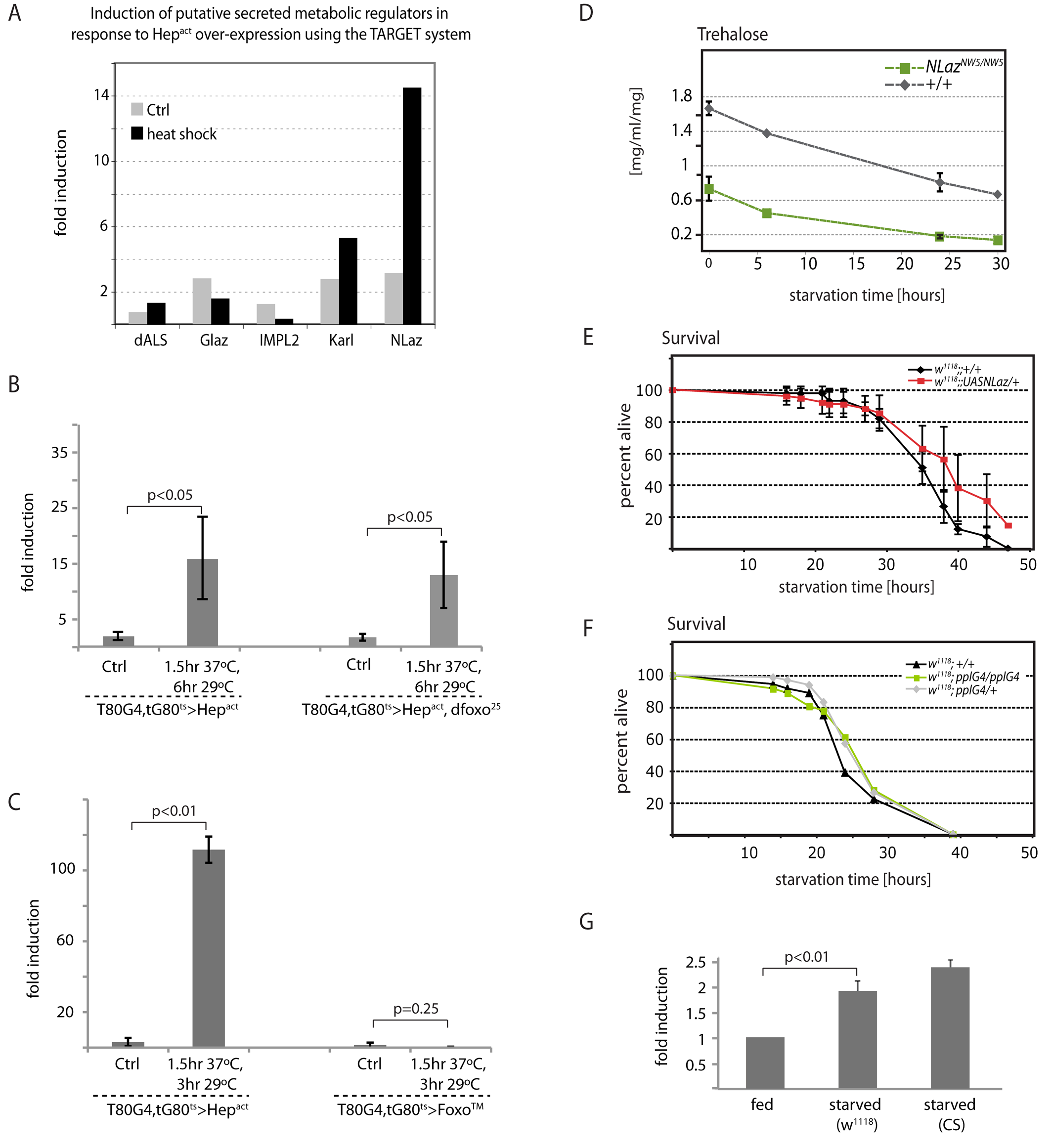

Supplement: Figure S2 — (A) Real time RT-PCR to measure changes in transcription of selected potential secreted metabolic regulators in response to JNK activation. The heat shock-inducible TARGET system was utilized. Average fold induction (between Hepact expressing and wild-type controls) of non-heat shocked (reared at 18 degrees) and heat shocked samples are shown. Actin5C expression was used for normalization. (B) Transcriptional response of NLaz to JNK activation is not dependent on Foxo. Real time RT-PCR to measure changes in transcription of NLaz in larvae in which Hepact is over-expressed using the TARGET system. Reducing the Foxo genedose (dfoxo25 is a loss-of-function allele of dfoxo) does not affect the level of NLaz induction. (C) Over-expression of constitutively active Foxo (FoxoTM) is not sufficient to induce NLaz transcription. (D) Trehalose content of NLazNW5/NW5 compared to isogenic wild-type controls. (E) Flies carrying the UAS-NLaz transgene in a wild-type background are not starvation sensitive. Sibling populations of progeny from crosses between w1118;; UAS-NLaz/+ to w1118 are shown (w1118;; UAS-NLaz/+; n = 76, w1118;; +/+; n = 72). (F) Flies carrying the ppl-Gal4 transgene in a wild-type background are not starvation sensitive. Sibling populations of progeny from crosses between w1118; pplG4/+ to w1118; pplG4/+ are shown (w1118; pplG4/+; n = 66, w1118; pplG4/pplG4; n = 36; w1118; +/+; n = 36). (G) NLaz is induced in response to starvation in wild-types flies. NLaz transcript levels in adults of two wild-type strains (w1118 and CantonS) were measured by qRT-PCR. Prolonged (20 hr) starvation results in moderate increase of NLaz transcript. (0.5 MB TIF) [file pgen.1000460.s002.tif]

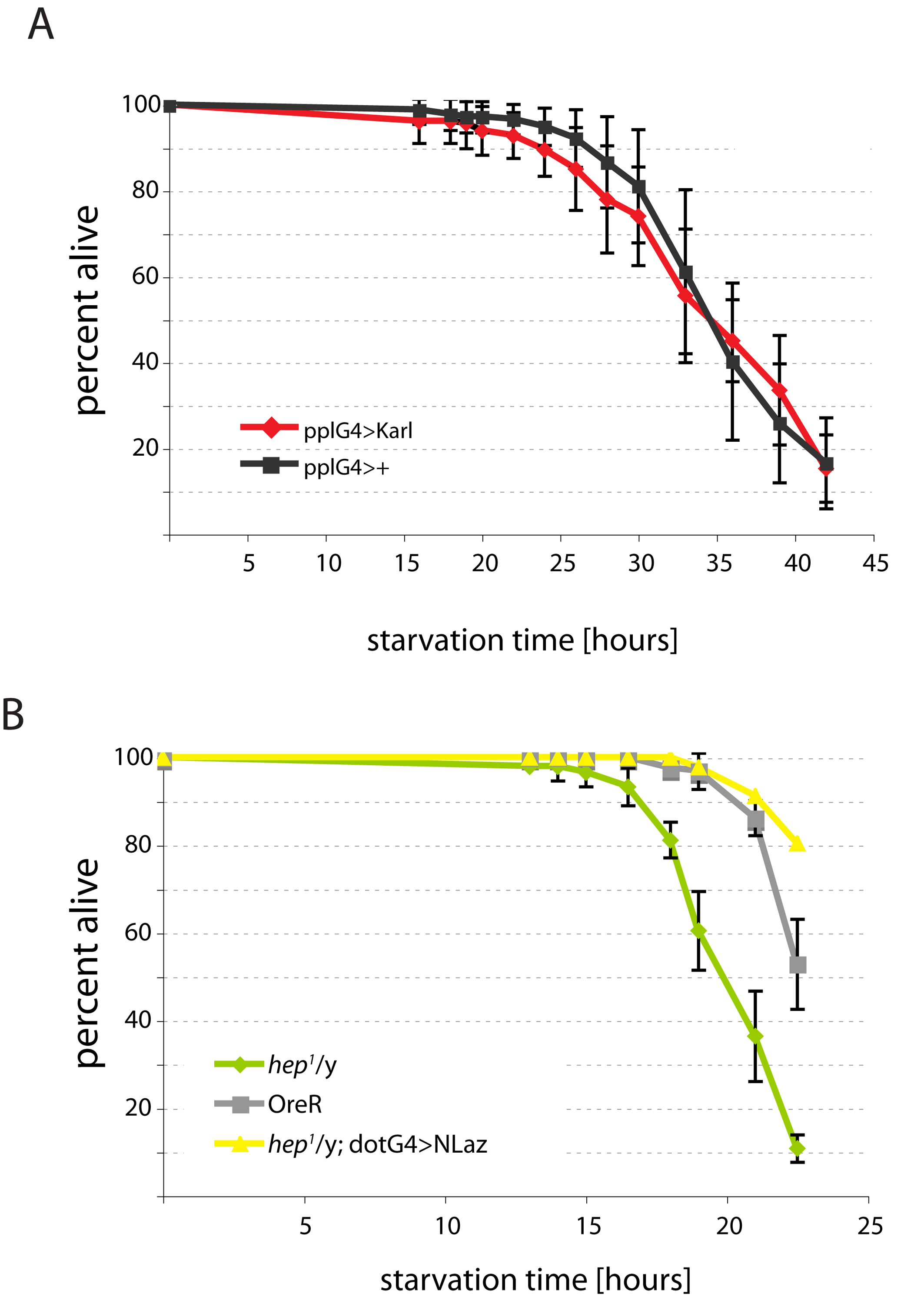

Supplement: Figure S3 — (A, B) Percent survival in response to dry starvation. Genotypes: (A) pplG4/+, n = 181, pplG4/+;UASKarl/+, n = 181. (B) hep1/y, n = 134, +/+, n = 130, hep1/y;DorothyG4/+;UASNLaz/+, n = 46. (0.4 MB TIF) [file pgen.1000460.s003.tif]

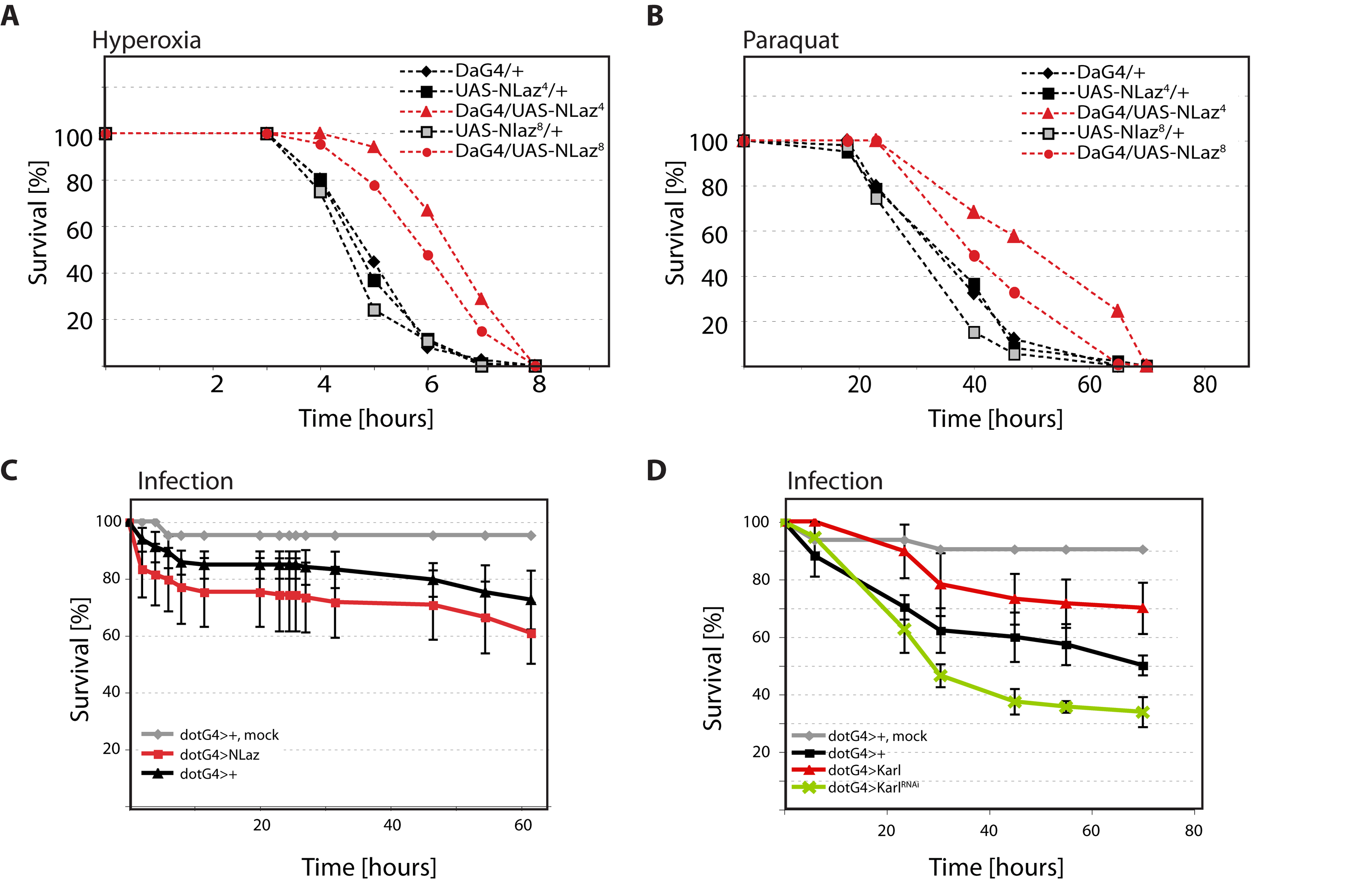

Supplement: Figure S4 — (A) Over-expression of NLaz enhances resistance to hyperoxia. Overexpressing UAS-NLaz4 and UAS-NLaz8, using DaG4 as a ubiquitous driver protects from 100% oxygen-induced mortality. DaG4/+, n = 157; UAS-NLaz4/+, n = 106; DaG4/UAS-NLaz4, n = 84; UAS-NLaz8/+, n = 104; DaG4/UAS-NLaz8, n = 107. Log rank test for UAS-NLaz4: p<0.001. Log rank test for UAS-NLaz8: p<0.001. (B) Overexpressing UAS-NLaz using DaG4 protects from paraquat-induced mortality. UAS-NLaz4 and UAS-NLaz8 are independent insertion lines of the same construct. DaG4/+, n = 118; UAS-NLaz4/+, n = 102; DaG4/UAS-NLaz4, n = 132; UAS-NLaz8/+, n = 94; DaG4/UAS-NLaz8, n = 116. Log rank test comparing UAS-NLaz4 and DaG4/UAS-NLaz4: p<0.001. Log rank test for UAS-NLaz8: p<0.001. (C, D) Percent survival in response to infection with E. faecalis. Genotypes: (C). DorothyG4/+ mock, n = 21, DorothyG4/+, n = 111; DorothyG4/+;UASNLaz/+, n = 108. (D) DorothyG4/+, mock, n = 31; DorothyG4/+, n = 84; DorothyG4/+; UASKarl/+, n = 60, DorothyG4/+;pWizKarl/+, n = 56. Only initial mortality was recorded here, as flies that escape initial mortality live for at least another 2–3 weeks. (0.6 MB TIF) [file pgen.1000460.s004.tif]

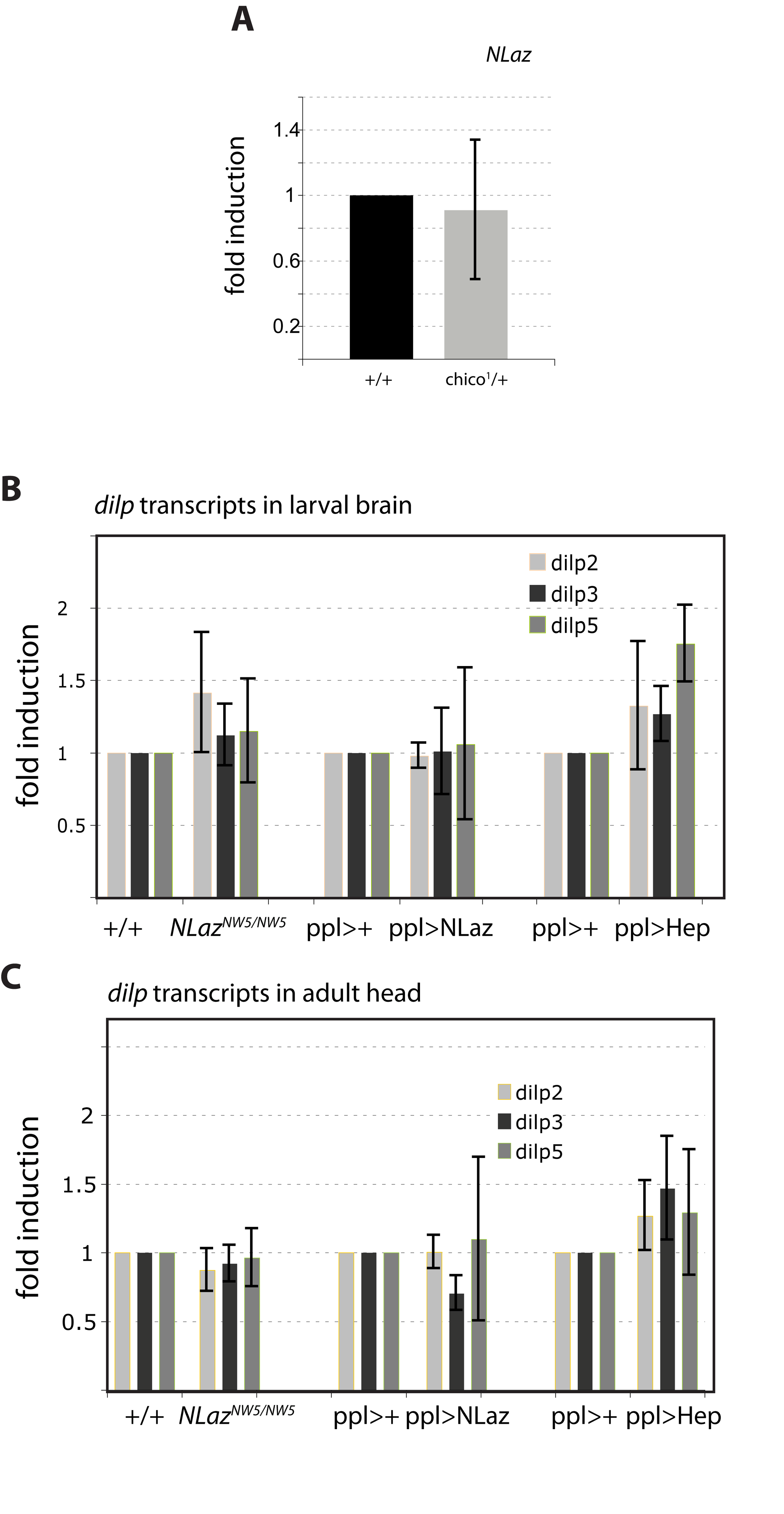

Supplement: Figure S5 — (A) Real time RT-PCR demonstrates that NLaz transcript levels are unchanged in chico1 heterozygous mutants. (B) Real time RT-PCR measuring levels of dilp2, dilp3, and dilp5 in cDNA prepared from dissected larval brains. Larval genotypes were as follows: +/+; NLazNW5/NW5; pplG4/+; pplG4/+;UASNLaz/+; pplG4/UASHep. Transcript levels were normalized to Actin5C. (C) Real time RT-PCR measuring levels of dilp2, dilp3, and dilp5 in adult heads from flies of the following genotypes: +/+, NLazNW5/NW5 pplG4/+, pplG4/+;UASNLaz/+, pplG4/UASHep. All transcript levels are normalized to Actin5C. (0.6 MB TIF) [file pgen.1000460.s005.tif]

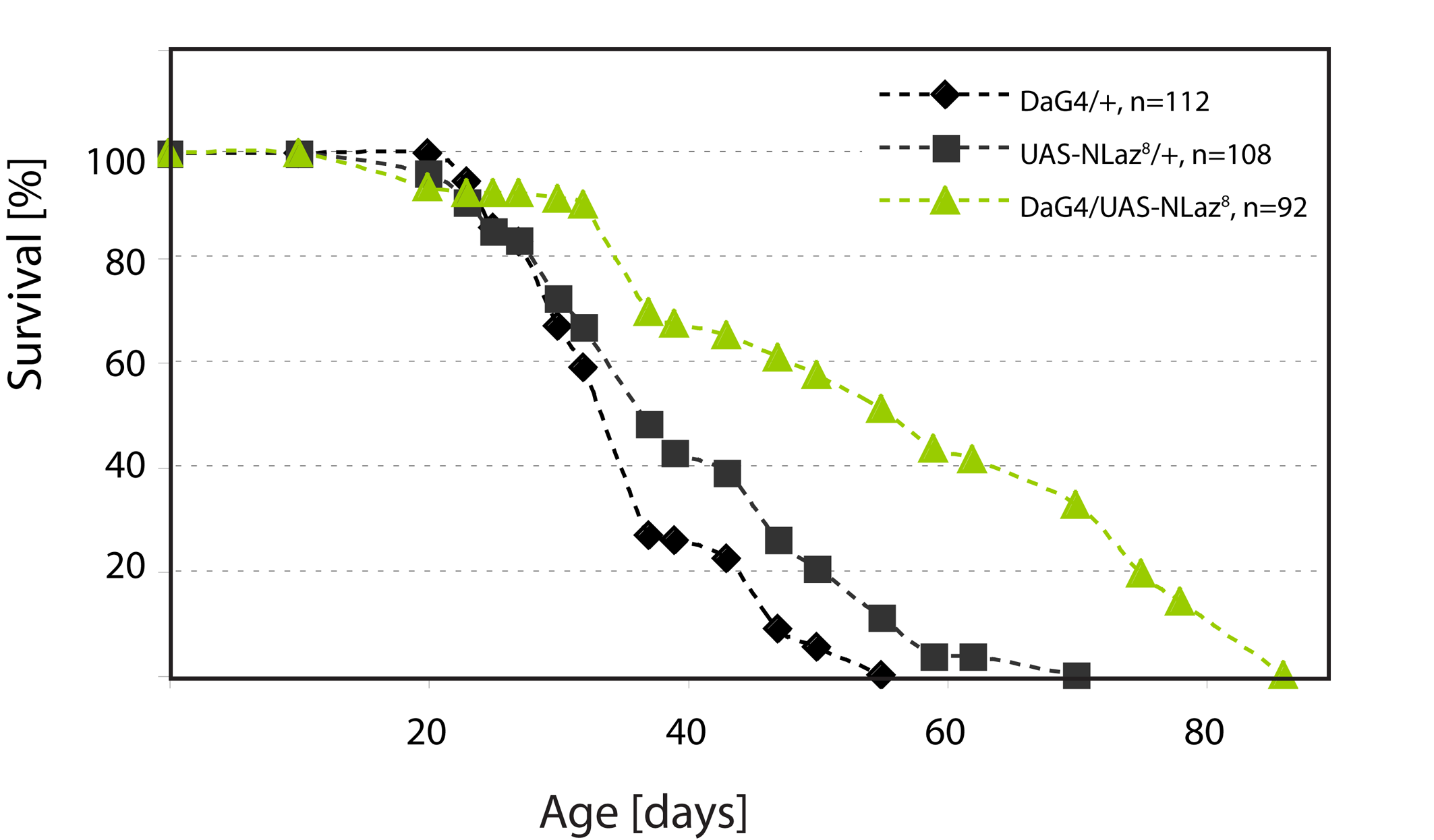

Supplement: Figure S6 — NLaz over-expression from an alternative transgenic line promotes longevity. Overexpressing UAS-NLaz8, using DaG4 as a ubiquitous driver, increases mean and maximum lifespans in normal conditions.UAS-NLaz8/+, n = 108; DaG4/UAS-NLaz8, n = 92. Log-rank test: p<0.001. (0.2 MB TIF) [file pgen.1000460.s006.tif]

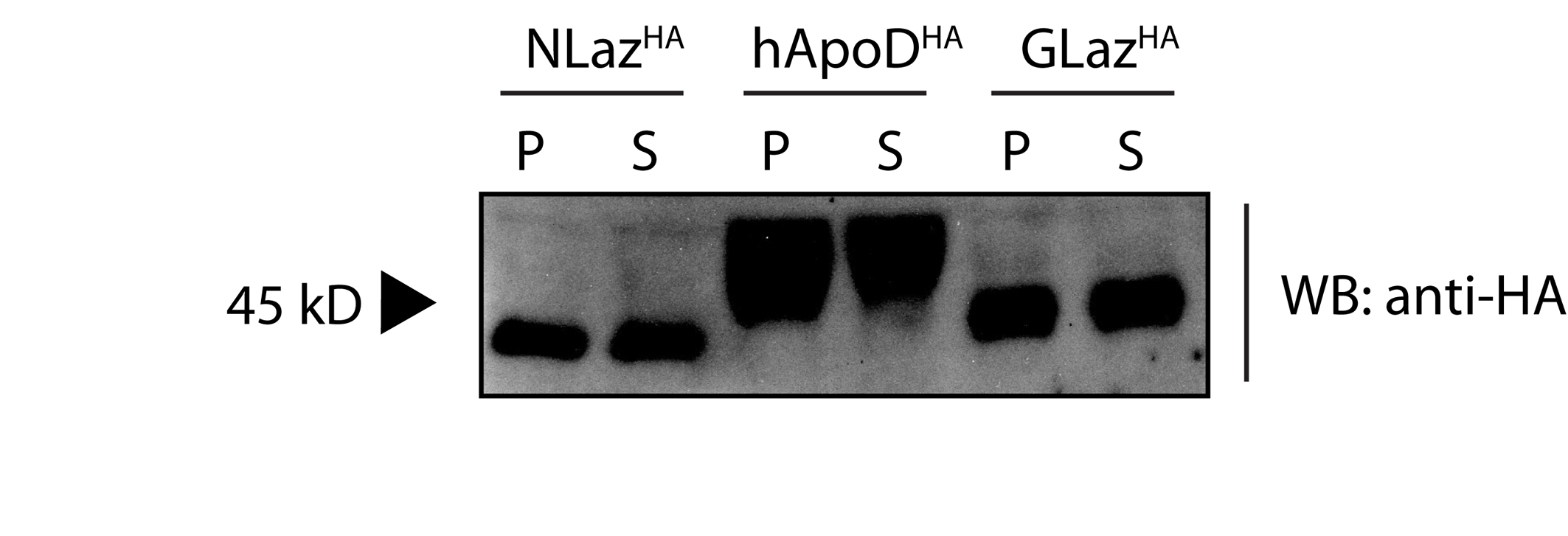

Supplement: Figure S7 — NLaz is secreted. HA-tagged NLaz (lanes 1 and 2) can be detected in the medium of S2 cells after 6 hrs of conditioning. Cell pellet (P) and supernatant (S) are shown. Related lipocalins are also secreted: human ApoD (lanes 3 and 4) and Drosophila GLaz (lanes 5 and 6). (0.3 MB TIF) [file pgen.1000460.s007.tif]

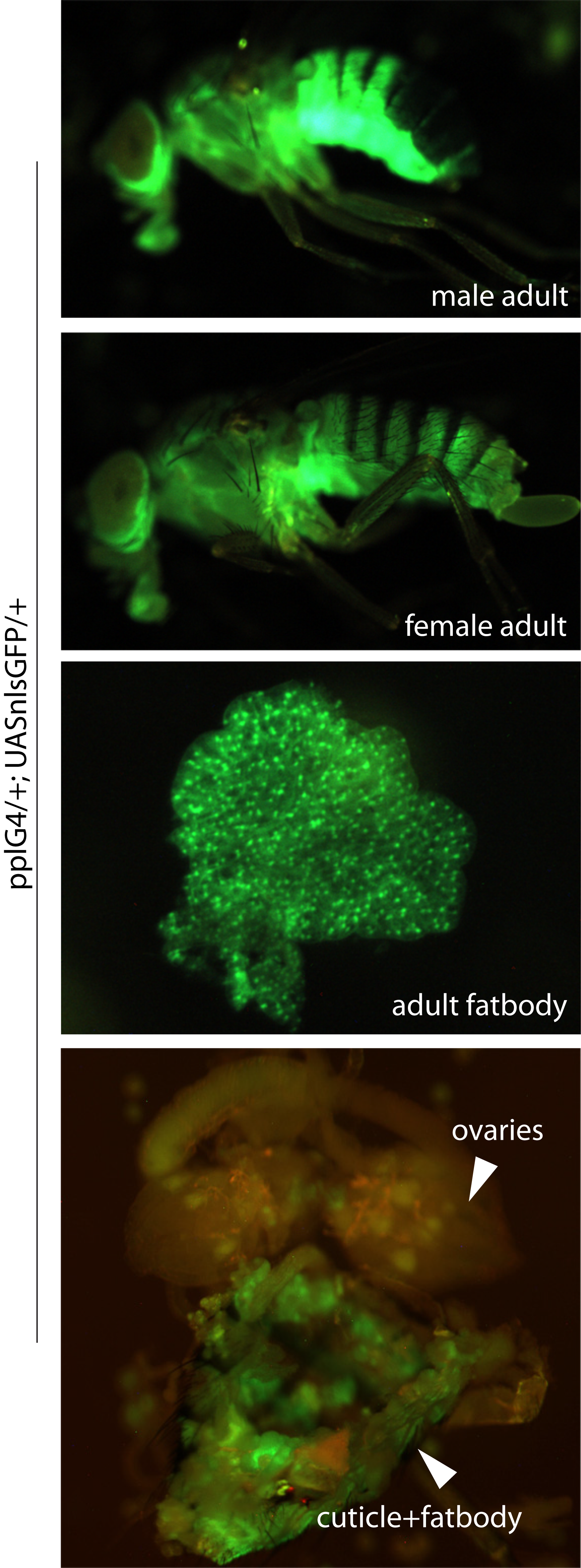

Supplement: Figure S8 — pplG4 is active in the adult fatbody. GFP fluorescence can be observed throughout the body of male and female adult flies when pplG4 is used to drive UAS-nlsGFP expression. This signal is derived from the head and abdominal fatbodies. Dissected abdominal fatbody is shown in the third panel. Ppl does not drive expression in ovaries (compare fluorescence in fatbody attached to cuticle and ovaries). (6.0 MB TIF) [file pgen.1000460.s008.tif]

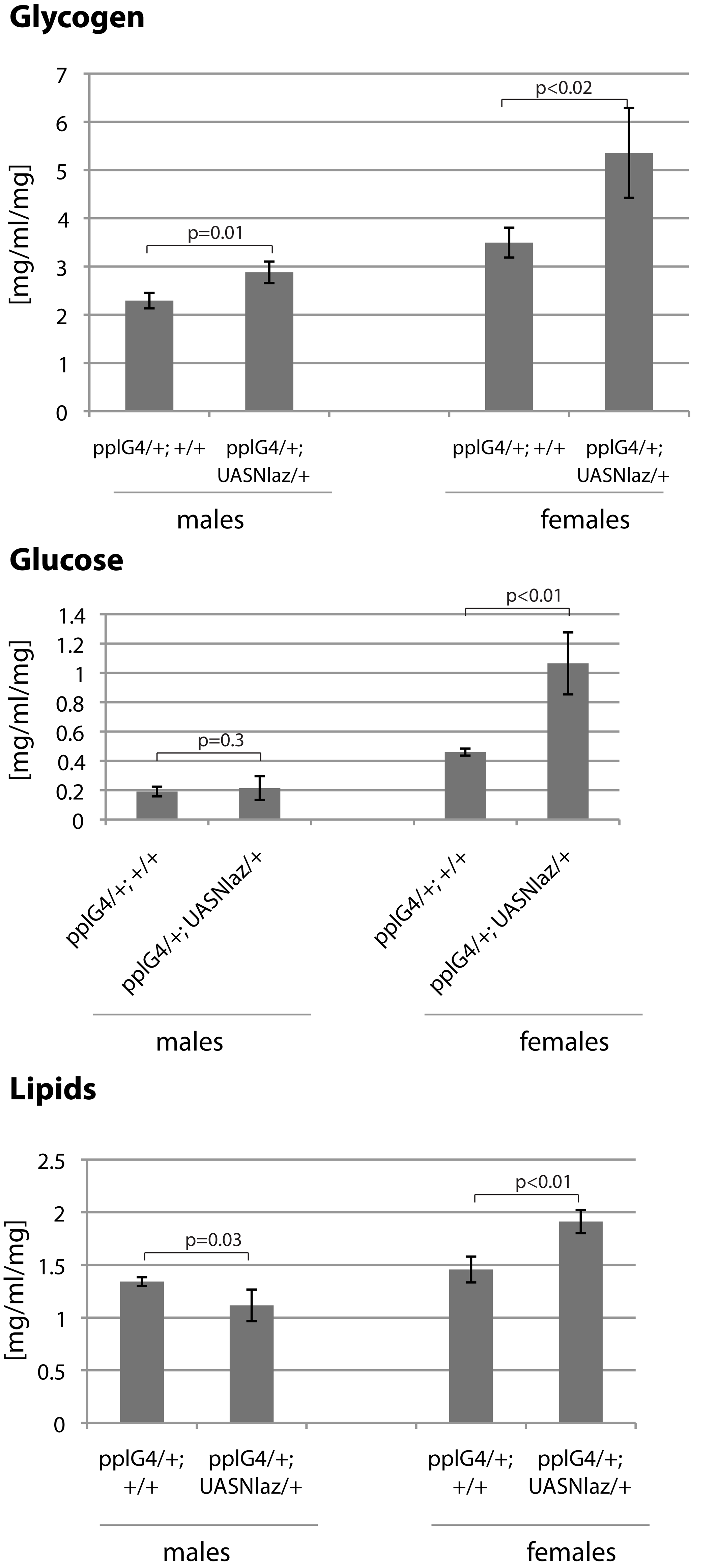

Supplement: Figure S9 — Overexpression of NLaz affects metabolites in males and females. Glucose, Glycogen and Lipid levels in adult flies over-expressing NLaz under the control of pplGal4. Flies were reared at 25°C on normal food and metabolites were measured at 5 days of age. (0.4 MB TIF) [file pgen.1000460.s009.tif]
